# A chemical avenue to manipulate field-reentrant superconducting competition in infinite-layer nickelates

**DOI:** 10.1093/nsr/nwag457

**Published:** 2026-07-27

**Authors:** Haowen Han, Yusong Zhao, Yi Bian, Tong Ma, Wenlong Yang, Shaohua Yang, Binghui Ge, Hongliang Dong, Chuanying Xi, Ze Wang, Nuofu Chen, Tian Shang, Toni Shiroka, Zaher Salman, Jia-Cai Nie, Ho-Kwang Mao, Jikun Chen

**Affiliations:** Beijing Advanced Innovation Center for Materials Genome Engineering, School of Materials Science and Engineering, University of Science and Technology Beijing, Beijing 100083, China; Beijing Advanced Innovation Center for Materials Genome Engineering, School of Materials Science and Engineering, University of Science and Technology Beijing, Beijing 100083, China; Beijing Advanced Innovation Center for Materials Genome Engineering, School of Materials Science and Engineering, University of Science and Technology Beijing, Beijing 100083, China; Beijing Advanced Innovation Center for Materials Genome Engineering, School of Materials Science and Engineering, University of Science and Technology Beijing, Beijing 100083, China; School of Physics and Astronomy, Beijing Normal University, Beijing 100875, China; Institute of Physical Science and Information Technology, Anhui University, Hefei 230601, China; Institute of Physical Science and Information Technology, Anhui University, Hefei 230601, China; Nanoscale and Compression Physics (NCP) Group, Center for High Pressure Science and Technology Advanced Research, Shanghai 201203, China; Shanghai Key Laboratory of Material Frontiers Research in Extreme Environments, Institute for Shanghai Advanced Research in Physical Sciences, Shanghai 201203, China; Anhui Key Laboratory of Low-Energy Quantum Materials and Devices, High Magnetic Field Laboratory, HFIPS, Chinese Academy of Sciences, Hefei 230031, China; Anhui Key Laboratory of Low-Energy Quantum Materials and Devices, High Magnetic Field Laboratory, HFIPS, Chinese Academy of Sciences, Hefei 230031, China; School of Renewable Energy, North China Electric Power University, Beijing 102206, China; Key Laboratory of Polar Materials and Devices (MOE), School of Physics and Electronic Science, East China Normal University, Shanghai 200241, China; Laboratory for Muon Spin Spectroscopy, PSI Center for Neutron and Muon Sciences, Paul Scherrer Institute, Villigen PSI 5232, Switzerland; Laboratory for Muon Spin Spectroscopy, PSI Center for Neutron and Muon Sciences, Paul Scherrer Institute, Villigen PSI 5232, Switzerland; School of Physics and Astronomy, Beijing Normal University, Beijing 100875, China; Key Laboratory of Multiscale Spin Physics, Ministry of Education, Beijing Normal University, Beijing 100875, China; Nanoscale and Compression Physics (NCP) Group, Center for High Pressure Science and Technology Advanced Research, Shanghai 201203, China; Shanghai Key Laboratory of Material Frontiers Research in Extreme Environments, Institute for Shanghai Advanced Research in Physical Sciences, Shanghai 201203, China; Beijing Advanced Innovation Center for Materials Genome Engineering, School of Materials Science and Engineering, University of Science and Technology Beijing, Beijing 100083, China

**Keywords:** superconductor, nickelates, reentrant superconductivity

## Abstract

Recently, preliminary magnetic-field-reentrant superconductivity manifested in high-temperature (*T*_c_) Eu-doped infinite-layer (IL) nickelates, beyond analogous discoveries exclusively in low-*T*_c_ systems. This evokes intriguing fundamental issues about potential quantum-phase boundary and criticality between unconventional superconductivity and field-reentrant one, which are inexplicable owing to formidable challenges in growing IL nickelates toward later-series rare-earths. Herein, we open up chemical avenues to enable effective growth of (*RE*_1__−_*_y_RE'_y_*)_1__−_*_x_*Eu*_x_*NiO_2_ (*RE*/*RE'*: Pr, Nd, Sm, Gd, Dy), giving rise to discoveries of *RE*-4*f*-related quantum competition between high-*T*_c_ and reentrant superconductivity. Robust magnetic-field-reentrant superconductivity with uniaxial anisotropy is observed at superconducting dome boundaries, stemming from Eu^2+^-4*f* ^7^-associated competition between magnetic-fluctuation-promoted pairing and exchange-field interactions. Their quantum criticality is further modulable via *RE*(*RE*′) magnetism, which either reinforces reentrancy or elevates *T*_c_ (∼40.1 K) with more robust critical current density (∼266 kA/cm^2^ at 2 K) beyond Sr-/Ca-doped counterparts. Our synthetic route enables the establishment of an ideal platform via IL nickelates for studying 4*f*-related unconventional superconductivity and quantum criticality.

## Teaser

Megapascal-level high-oxygen-pressure chemical deposition enables high-effectivity growth of Eu-doped IL nickelates for heavier rare-earths, uncovering Eu-modulated competition between field-reentrant and high-Tc superconductivity.

## INTRODUCTION

The participance of *f* electrons in ferromagnetic coupling [1], Kondo lattice [1,2] and antiferromagnetic exchange-mediated Cooper pairing [3,4], largely enriches the superconducting phase diagram, also giving rise to magnetic-field-reentrant superconductivity [5]. Recently, preliminary sign of reentrant superconductivity was observed via introducing the half-filled Eu^2+^ (4*f* ^7^) as hole dopant for infinite-layer (IL) nickelates [6–8], which belongs to a new family of high temperature superconductor [9] with *T*_c_ near ∼40 K [10]. This is in stark contrast to the analogous earlier discoveries in exclusive low-*T*_c_ systems, such as UTe_2_ [5,11,12], URhGe [13], Eu-containing Chevrel phase compounds [14,15], λ-(BETS)_2_FeCl_4_ [16,17] and moiré graphene [18]. It unveils a previously unexplored dimension associated with the rare-earth (*RE*) 4*f-*orbital and/or magnetic effects that may exert a substantial effect on superconducting phase diagram of nickelates, beyond their conventional ionic size effects [19,20]. This observation further coincides with the distinct magnitude and anisotropy in the superconducting upper critical field as observed for IL nickelates with various magnetic contributions by the *RE*-4*f* moments [21]. Also, it is more intriguing to note the generally elevated *T*_c_ of ambient pressured nickelate superconductor via substituting their *RE* composition toward later

lanthanide series, as presently valid for not only IL nickelate [10,21,22], but also thin-film-layered perovskite nickelates [6,23–26]. In light of the prevailing trajectory of nickelate superconductors, venturing into heavier *RE* to map potential 4*f*-orbital effects on superconducting phase diagram offers the prospect of groundbreaking superconductivities and fundamental elucidations of underlying mechanisms.

Nevertheless, the key challenge in exploring nickelate superconductors toward heavier *RE* lies in their material growths [10,22,27]. Presently, successful growths of IL nickelates manifesting superconductivity were exclusively via vacuum epitaxy of perovskite nickelates precursors followed by topotactic reduction, limited to light *RE* prior to Eu [27]. Owing to the lanthanide contraction, introducing heavier *RE* (e.g. behind Eu) with smaller ionic radius of *RE* (*r*_RE_) into perovskite nickelates is thermodynamically more difficult. This is because a more distorted NiO_6_ octahedra elevates the formation free energy (Δ*G*) [19] of perovskite nickelates toward more positive magnitude, which is hardly stabilizable by coherent lattice with the substrate. Further obstacle is from the restriction in compositing the alkaline-earth (*AE*) hole dopants within perovskite nickelates, since conventional chemical process is incapable to form Ni^4+^ in perovskites as major constituents even at extremely high *p*_O2_ of approximately gigapascal level [20,28]. Thus, perovskite nickelates containing *AE* constituents were likely to be heterogeneously formed via plasma or atomic beam interplays with the substrate, contingent upon precise and narrowly defined experimental window [10,22,27]. From these perspectives, the present strategy for growing IL nickelates is insufficient to support further explorations pertaining to heavier *RE*, despite its strong likelihood to realize higher *T*_c_ and/or unconventional superconductivity, e.g. field reentry [6–8].

Here, we open up a simple high-*p*_O2_-assisted chemical avenue for growing IL nickelate superconductors toward heavier *RE* constituents with effectiveness in *RE* substitutions, grounded in which their 4*f*-orbital-relevant field-reentrant superconducting phase diagram is elucidated. Robust uniaxially anisotropic superconducting reentrancy was validated to emerge at the quantum phase boundaries of the superconducting dome for both Nd_1__−_*_x_*Eu*_x_*NiO_2_ and Pr_1__−_*_x_*Eu*_x_*NiO_2_ systems. Their quantum criticality was further modulated via introducing *RE'* within (Nd_1__−_*_y_RE'_y_*)_1__−_*_x_*Eu*_x_*NiO_2_ (*RE'*: Pr, Sm, Gd, Dy and Sm_1/2_Pr_1/2_) to adjust the magnitude of exchange field associated with *RE* magnetism. We highlight the further elevation in *T*_c_ and critical current density (*J*_c_) for the Eu-doped IL nickelates, exceeding previous layer spacing expectations developed from their counterpart systems doped by Sr/Ca. The root cause was further elucidated, combining characterization of pairing strength via magneto-transport and localized magnetism via low-energy muon-spin spectroscopy (LE-*μ*SR). Our work unveils the pivotal role of magnetic fluctuations with close associations with *RE*-4*f* in modulating the quantum criticality and pairing strength, conventional Bardeen-Cooper-Schrieffer (BCS) theory. Also, our megapascal (MPa)-*p*_O2_-assisted chemical synthetic route sheds a light on the capability to apply IL nickelates as coated conductors analogous to high-*T*_c_ cuprates [29].

## RESULTS AND DISCUSSIONS

### A MPa-high *p*_O2_-assisted chemical avenue enables effective growth of IL nickelates

To reduce the positive Δ*G* of perovskite nickelates contracting with lanthanide contractions, we exploit an MPa-high *p*_O2_-assisted chemical strategy for thin film growth of IL nickelate, illustrated in Fig. S1a. In brief, the chemical precursors of *RE*(NO_3_)_3_ and Ni(CH_3_COO)_2_ were dissolved in ethylene glycol monomethyl ether (EGME), and spin coated on an NdGaO_3_ (110) substrate. The spin-coated films were crystallized into the perovskite precursor film by annealing at 700–900°C under a high *p*_O2_ within 1–20 MPa. Afterward, the perovskite nickelates were transformed into IL via soft chemical topotactic reduction based on CaH_2_ co-anneals at a temperature between 260 and 320°C for 0.5–4 h. Instead of using conventional *AE* elements (e.g. Sr or Ca) [30,31], herein the hole doping was realized via partially substituting their *RE* constituents by Eu, which displays variable valance state from +3 in perovskites toward +2 upon topotactic reduction [22]. From the thermodynamic perspective, the metastable perovskite nickelates precursors containing later-series *RE* composition are preferentially stabilized at a high-*p*_O2_ within 10^0^ and 10^2^ MPa [19,20], as indicated by their equilibrium phase chart in Fig. 1a (see more discussions in Figs S1–S3). Hence, our strategy is capable to introduce later-series *RE* further behind Eu into IL nickelates, while the *RE* constituent is simply and flexibly modulable manipulating the chemical solutions for spin coating.

**Figure 1.**
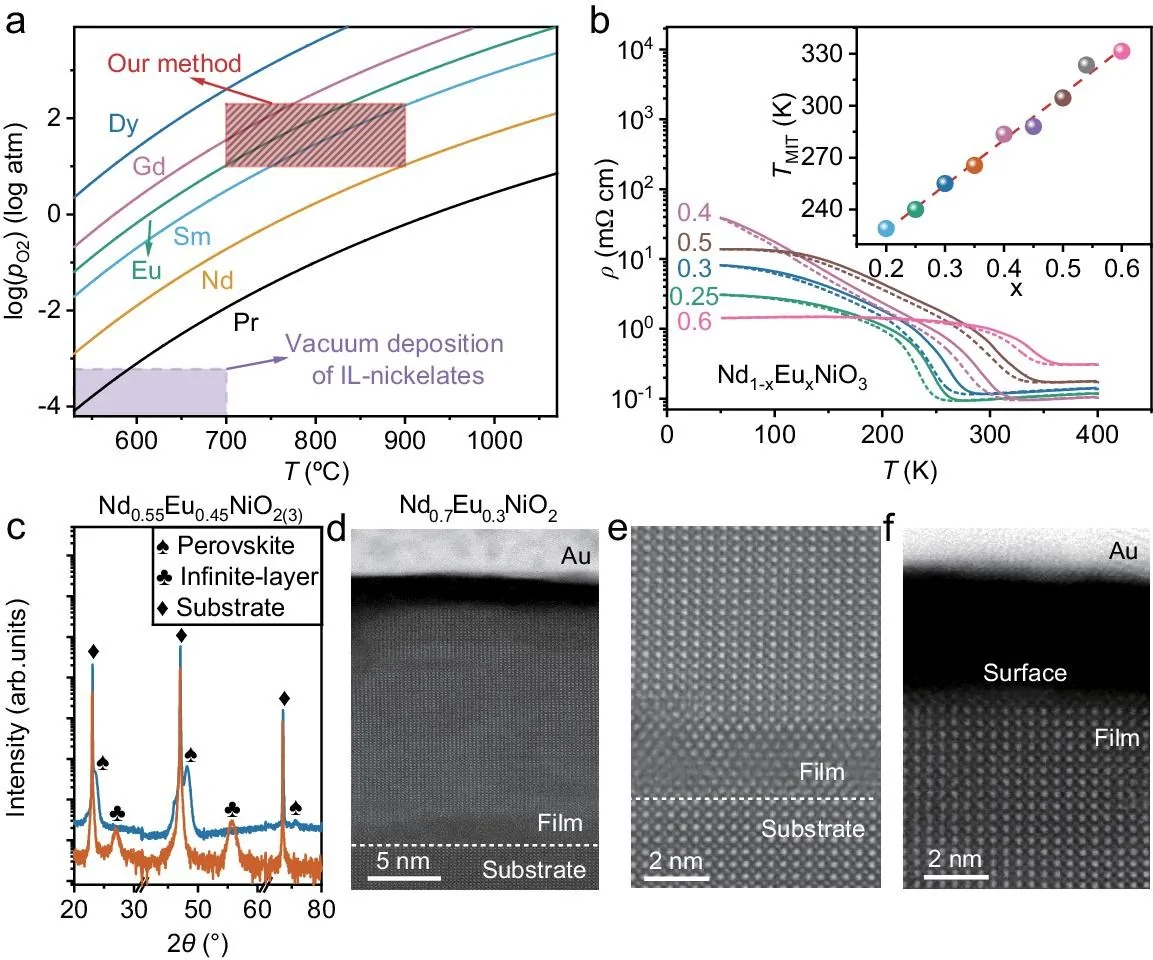
High-oxygen-pressure-assisted chemical avenue for IL nickelates. (a) The pressure-temperature (*p*–*T*) phase diagram for the formation of perovskite nickelates with different *RE* elements under high oxygen pressure is depicted. The upper shaded region outlines the range of synthesis conditions encompassed by our method, with the hatched area indicating the specific parameters employed in this work. The lower shaded region represents the typical synthesis conditions for vacuum deposition of perovskite nickelate thin films. (b) Temperature dependence of resistivity measured for Nd_1__−__x_Eu_x_NiO_3_ thin films with various Eu-substituting compositions (*x*), convincing their abrupt metal–insulator transition behavior. The inset shows their metal–insulator transition temperature (*T*_MIT_) versus *x*. (c) The XRD patterns for Nd_0.55_Eu_0.45_NiO_3_ perovskite precursor and the Nd_0.55_Eu_0.45_NiO_2_ IL. (d) Cross-sectional HAADF-STEM image of Nd_0.7_Eu_0.3_NiO_2_ on NdGaO_3_(110). (e and f) Magnified HAADF-STEM images of (e) the interfacial region and (f) the film surface.

Efficient growth of IL nickelates is first exemplified by Nd_1__−_*_x_*Eu*_x_*NiO_2_, covering a large variety in Eu compositions. Via MPa-high *p*_O2_ anneals, the metastable Nd_1__−_*_x_*Eu*_x_*NiO_3_ is well stabilized, as indicated by their abrupt metal–insulator transitions (see Fig. 1b and Fig. S4) [32,33]. The accuracy in the stoichiometry controls for Eu is convinced by the linear increase in metal–insulator transition temperature (*T*_MIT_) with *x* (or average *r*_RE_) in consistency with the previous reports [19,20], as shown by the inset of Fig. 1b. More details in determination of *T*_MIT_ are shown in Fig. S5. As shown in Fig. 1c, the X-ray diffraction (XRD) patterns of the Nd_0.55_Eu_0.45_NiO_3_ demonstrate their oriented perovskite crystal structure, which transformed to the Nd_0.55_Eu_0.45_NiO_2_ ILs after the topotactic reduction (see more XRD results in Fig. S6). The reduction process may result in reduced crystallinity and interfacial coherency indicated by the broadened XRD peak, as also observed previously in vacuum deposited IL nickelates [9,10]. The resultant variation in electronic structure, e.g. from Ni^3+^ to Ni^1+^, is further confirmed by synchrotron-based X-ray absorption spectroscopy (XAS) analysis, as shown in Fig. S7. Figure 1d–f shows the archetypal cross-section morphology for Nd_1__−_*_x_*Eu*_x_*NiO_2_/NdGaO_3_ (110) as probed by high-angle annular dark field (HAADF), where a larger thickness of ∼20 nm is observed compared with the vacuum-deposited ones (e.g. 6–8 nm) [9,10,22,34]. More representative microscopic morphologies and elementary distributions are demonstrated in Figs S8–S11. It is also worth noticing that the Nd_1__−_*_x_*Eu*_x_*NiO_3_ precursor is coherently grown on the NdGaO_3_ substrate (see Fig. S12), while the topotactic reduction slightly reduces the crystallinity and coherency of the interface.

### Tuning the Eu-related competition between reentrant and high-*T*_c_ superconductivity

Robust superconducting behaviors are observed for Nd_1__−_*_x_*Eu*_x_*NiO_2_ with *x* = 0.25–0.55, with the highest *T*_c,onset_, *T*_c_,_50%_ and *T*_c,zero_ emerged at optimum Eu constituents (*x* = 0.35–0.45) reaching ∼37.6, ∼27.3 and ∼22.2 K, respectively. The temperature dependencies in resistivity (*ρ–T*) of archetypal Nd_1__−_*_x_*Eu*_x_*NiO_2_ are shown in Fig. 2a for optimum doping (*x* = 0.45), underdoping (*x* = 0.3) and overdoping (*x* = 0.52), respectively; while the *ρ–T* tendencies for more Eu-doping constituents are provided in Fig. S13. The optimum-doped Nd_1__−_*_x_*Eu*_x_*NiO_2_ (*x* = 0.35–0.45) manifest expected high-*T*_c_ superconductivity that is monotonically suppressed by magnetic fields up to 35 T with no signature of reentrance (see Fig. S14). Figure S13J and K further demonstrates its high magnitude of *J*_c_ reaching 266 kA/cm^2^ at 2 K, exceeding previous vacuum-deposited Nd-Sr and Nd-Eu systems at the same temperature (e.g. *J*_c_ = 170 kA/cm^2^ for Nd_0.8_Sr_0.2_NiO_2_ [9] and *J*_c_ = 237 kA/cm^2^ for Nd_0.65_Eu_0.35_NiO_2_ [8]). Only limited reduction in the magnitudes of *J*_c_ is observed, even imparting cross-plane magnetic fields up to 9 T. In contrast, the magnetic field reentrant superconducting behaviors reaching zero resistance are observed for Nd_1__−_*_x_*Eu*_x_*NiO_2_ at both underdoped (*x* = 0.2–0.3) and overdoped (*x* = 0.52–0.6) regions. This is demonstrated by their *ρ–B* tendency in the insets of Fig. 2a (also Fig. S14), and also the crossing in their *ρ–T* tendencies as measured under different magnetic fields in Fig. S15.

**Figure 2.**
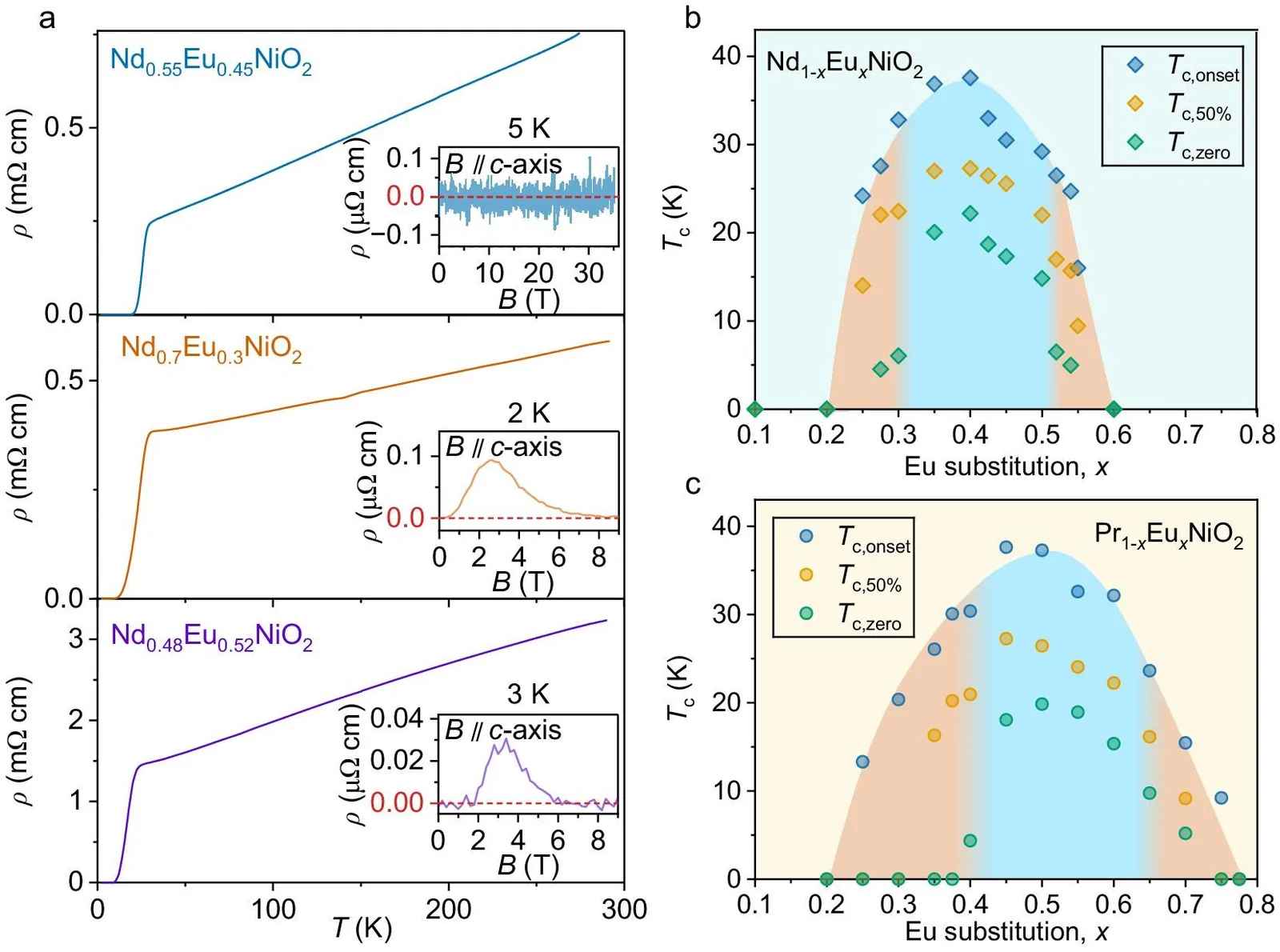
Discovery of field-reentrant superconductivity at the quantum boundaries of superconducting dome. (a) The temperature-dependent resistivity (*ρ*–*T*) measured for: the optimally doped Nd_0.55_Eu_0.45_NiO_2_, with the inset showing the corresponding magnetic-field-dependent resistivity (*ρ*–*B*) at 5 K that demonstrated conventional superconductivity; the underdoped Nd_0.7_Eu_0.3_NiO_2_, with the inset showing *ρ*–*B* at 2 K that demonstrates reentrance; the overdoped Nd_0.48_Eu_0.52_NiO_2_, with the inset showing *ρ*–*B* curve at 3 K that demonstrates reentrance. (b and c) Superconducting phase diagram for (b) Nd_1__−__x_Eu_x_NiO_2_ and (c) Pr_1__−__x_Eu_x_NiO_2_ plotted as a function of Eu substituting composition, *x*. The central shaded region represents high-T*c* superconductivity without reentry, whereas the two flanking shaded regions represent the emergence of magnetic-field-reentrant superconductivity. The onset critical temperature (*T*_c,onset_) is corresponding to the temperature where the electrical resistivity begins to deviate notably from its normal-state behavior. The midpoint transition temperature (*T*_c, 50%_) represents the temperature where the resistivity drops to 50% of its normal-state value under zero magnetic field (typically referenced to the value at *T*_c,onset_). The zero-resistance temperature (*T*_c,zero_) is defined as the temperature where the electrical resistivity drops below the experimental detection limit. The dome is established from samples at more Eu-substituting compositions, as shown by Figs S13 and S16.

The field-reentrant superconductivity emerging at both the underdoped and overdoped boundaries also holds for Pr_1__−_*_x_*Eu*_x_*NiO_2_/NdGaO_3_ (110) and Nd_1__−_*_x_*Eu*_x_*NiO_2_/(LaAlO_3_)_0.3_(Sr_2_TaAlO_6_)_0.7_ (100), as demonstrated in Fig. S16. This indicates the competition between reentrant and high-*T*_c_ superconductivity within Eu-doped IL nickelates, giving rise to a more complicated superconducting phase diagram (*T*_c_–*x*) compared to the Sr/Ca-doped ones, as shown in Fig. 2c. We highlight that the superconducting reentry emerges exclusively at the superconducting boundaries, but is absent when the high-*T*_c_ superconductivity is robust at optimum doping. This unveils the previously underestimated tunability in the quantum criticality by *RE*-4*f* (particularly Eu-4*f* ^7^) [6], via the likelihood of a magnetic fluctuation modulated pairing in the neighborhoods of quantum phase transitions [11]. For example, in the underdoped region, the superconducting state has energy that is close to many competing states, such as charge order [35], spin order [36] and/or oxygen order [37]. In the overdoped region near a hidden quantum critical point, the system exhibits a transition from non-Fermi liquid to Fermi liquid behavior [30], triggering a sudden change in electronic state. Hence, potential *RE*-4*f*-modulated magnetic fluctuations and the suppression of superconductivity by the competing quantum states [34] may lead to the criticality between reentrant and high-*T*_c_ superconductivity [11,36].

Of particular note is the broader superconducting dome extending to larger Eu compositions in Pr_1__−_*_x_*Eu*_x_*NiO_2_ than in Nd_1__−_*_x_*Eu*_x_*NiO_2_, despite the similar magnitudes in their starting point (e.g. *x* = 0.2) and maximum *T*_c_, both of ∼37.6 K. Further X-ray photoemission spectroscopy analysis as shown in Fig. S18a–c indicates a higher Eu^2+^/Eu^3+^ ratio for Nd_1__−_*_x_*Eu*_x_*NiO_2_ (e.g. ∼1/1) compared to Pr_1__−_*_x_*Eu*_x_*NiO_2_ (e.g. ∼2/3). Hence, estimating their practical hole doping range contributed by Eu^2+^ indicates similarities to the previous reports for Nd_1__−_*_x_*Sr*_x_*NiO_2_ [30] and Pr_1__−_*_x_*Sr*_x_*NiO_2_ [38], as more clearly demonstrated in Fig. S17d and e. It is also worth noticing that in other contemporaneous report [6], the reentrant superconducting behavior of Sm_0.95__−_*_x_*Ca_0.05_Eu*_x_*NiO_2_ via vacuum growth emerges exclusively in the overdoped region, but not the underdoped one. Identifying the underlying reason requires further investigations, as there are fundamental distinctions in both the material composition and methodology of growth between the present work and Ref. [6]. One of the possibilities lies in the likelihood of the variation in superconducting boundaries via introducing Ca and/or Sm with smaller ionic radius compared to this work. Another possibility that cannot be completely excluded at this moment is potential extrinsic impacts from the microscopic defects or interfacial charge discontinuity, in particular when growing ILs on perovskites substrate. Although a decreasing tendency in cross-plane lattice parameter with *x* is indicated by the leftward shift in diffraction peaks of both Nd_1__−_*_x_*Eu*_x_*NiO_2_ and Pr_1__−_*_x_*Eu*_x_*NiO_2_ (see Fig. S6b–d), completely ruling out the above extrinsic impacts yet requires further targeted experimentation.

### Further modulations in the quantum criticality and *T*_c_ via multiple *RE* compositions

To further elucidate the role of *RE* in competition between high-*T*_c_ and reentrant superconductivity, we start with the matrix of optimum-doped Nd_1__−_*_x_*Eu*_x_*NiO_2_ (*x*: 0.35–0.45) without reentrancy and partially substitute Nd by *RE’* (e.g. Pr, Sm, Gd and Dy). The *RE'* substitution elicited corresponding changes in the *T*_MIT_ of (Nd_1__−_*_y_RE'_y_*)_0.65_Eu_0.35_NiO_3_ (see Fig. S18) and also lattice constants of (Nd_1__−_*_y_RE'_y_*)_0.65_Eu_0.35_NiO_2_ (see Fig. S19). Figure 3a (upper) shows the *ρ–T* tendencies for (Nd_0.9_Pr_0.1_)_0.65_Eu_0.35_NiO_2_, (Nd_0.9_Sm_0.1_)_0.65_Eu_0.35_NiO_2_ and (Nd_0.9_Dy_0.1_)_0.65_Eu_0.35_NiO_2_, where robust superconducting behaviors are observed, e.g. with *T*_c,zero_ of ∼20, 18 and 14 K, respectively. As their *ρ–B* tendencies are further demonstrated in the insets and also Fig. S20a–c, these samples display no field-reentrant behaviors. In contrast, the reentrant superconductivity emerges for (Nd_1__−_*_y_*Gd*_y_*)_0.65_Eu_0.35_NiO_2_ (*y* = 0.05, 0.1 and 0.2) as demonstrated in Fig. 3a (lower) and also the inset. Their *ρ–B* tendencies measured at more temperatures are further shown in Fig. S20d and e. Also, the Gd substitution results in lower *T*_c,zero_ compared to the ones substituted by other *RE'* (see Fig. S21).

**Figure 3.**
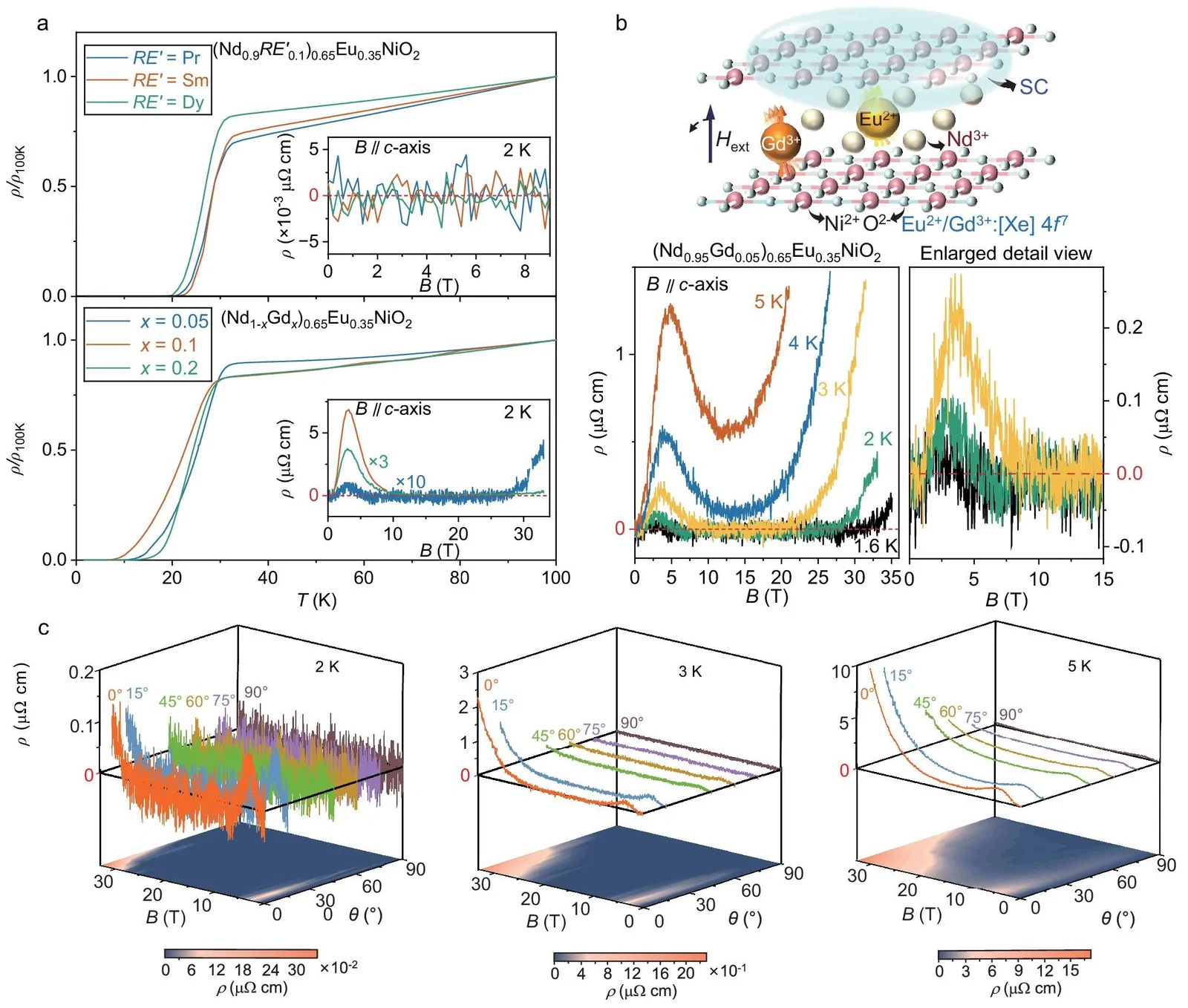
Further modification in criticality with field-reentrant superconductivity via introducing other *RE* substituents. (a) The normalized temperature-dependent resistivity (*ρ*–*T*) measured for (Nd_0.9_*RE'*_0.1_)_0.65_Eu_0.35_NiO_2_ (*RE'*=Pr, Sm and Dy), displaying robust conventional superconductivity. The inset shows their corresponding magnetic-field-dependent resistivity (*ρ*–*B*) measured at 2 K. The normalized *ρ–T* as measured for (Nd_1__−__*x*_Gd_*x*_)_0.65_Eu_0.35_NiO_2_ (*x* = 0.05, 0.1 and 0.2), showing clear field-reentrant superconductivity. The inset shows their corresponding *ρ*–*B* measured at 2 K. (b) Schematic illustration of the proposed mechanism: the 4*f* ^7^ moments of Gd^3+^ located at deeper energy level generate an internal exchange field (*H*_ex_) that partially compensates the external magnetic field (*H*_ext_) via the J–P effect [40], giving rise to reentrant superconductivity. *ρ*–*B* measurements of (Nd_0.95_Gd_0.05_)_0.65_Eu_0.35_NiO_2_ up to higher magnetic fields at different temperature. (c) Resistivity of (Nd_0.95_Gd_0.05_)_0.65_Eu_0.35_NiO_2_ measured for various angles between the external magnetic field and *c*-axis at: 2, 3 and 5 K. The bottom projection shows a resistivity map, where zero-resistance regions corresponding to reentrant superconductivity emerge at high fields when Hext is near the *c*-axis (*θ* ≈ 0°), revealing strong uniaxial anisotropy. In addition, elevating the temperature progressively suppresses the reentrant superconducting phase, constraining it within a narrow range of angles and fields.

To verify the zero resistance for the Gd-reinforced reentrant superconductivity and its secondary quench, we measured their *ρ–B* tendencies of (Nd_0.95_Gd_0.05_)_0.65_Eu_0.35_NiO_2_ up to higher magnetic fields at different temperatures, as shown in Fig. 3b. The reentrant superconductivity reaching zero resistances is observed from 5–10 T, 8–13 T and 10–15 T at 1.6, 2 and 3 K, respectively, afterward quenching at 30, 25 and 20 T. Further angular-dependent magneto-transport measurements via altering the angle (*θ*) between *H*_ext_ and *c*-axis indicate strong uniaxial cross-plane anisotropy in the superconducting reentrant behavior of (Nd_0.95_Gd_0.05_)_0.65_Eu_0.35_NiO_2_. Their resistivity is mapped as a function of both magnetic field strength and *θ* in Fig. 3c at 2, 3 and 5 K, where more separated regions associated with the low field and reentrant field superconductivity are observed when imparting *H*_ext_ along *c-*axis (*θ* = 0°). Also, elevating the temperature (e.g. from 2 to 5 K) results in markedly contraction in the reentrant region. This should be attributed to the higher *H*_c2_ along the *ab*-plane compared to the *c*-axis, stemming from the Eu-4*f*-related anisotropy in orbital depairing [21], as further discussed in Fig. S22. Similar phenomenon was also observed for as-grown (Nd_0.9_Gd_0.1_)_0.65_Eu_0.35_NiO_2_, Nd_0.48_Eu_0.52_NiO_2_ and Nd_0.46_Eu_0.54_NiO_2_, as demonstrated in Fig. S23. Hence, the Gd-reinforced reentrant superconductivity is in consistency to ones observed for Nd-Eu and Sm-Eu systems grown by both vacuum depositions [6–8].

The preferential superconducting reentrancy via introducing Gd over other *RE'* (e.g. Pr, Sm and Dy) within (Nd_1__−_*_y_RE'_y_*)_1__−_*_x_*Eu*_x_*NiO_2_ was also verified in other compositions (e.g. *x* = 0.35, *y* = 0.2; *x* = 0.45, *y* = 0.2; *x* = 0.5, *y* = 0.1), as demonstrated in Fig. S24. Instead of other proposed mechanisms (e.g. spin-triplet pairing [11] and metamagnetic criticality [39]), the Jaccarino–Peter (J–P) effect [40,41] was more likely to explain the magnetic-field-induced reentrant superconductivity for Eu-doped IL nickelates [6–8]. In sight of its half-filled 4*f* ^7^ among *RE*^3+^ (see details in Table S1) [42,43], introducing Gd^3+^ is expected to enhance the internal exchange field (*H*_ex_) to partially against the external magnetic field (*H*_ext_), as illustrated in Fig. 3c. In contrast, the Eu^2+^ (also with 4*f* ^7^) not only generates *H*_ex_, but also serves as hole dopant that simultaneously contributes to transports, giving rise to more complicated impact beyond simply J–P effect.

From a counterpoint compared to Gd, introducing *RE'* (e.g. Pr, Sm and Pr_1/2_Sm_1/2_) to reduce the magnetism of *RE* site within (Nd_1__−_*_y_RE'_y_*)_0.65_Eu_0.35_NiO_2_ sheds a light on further elevating *T*_c_ beyond the superconducting dome of the matrix Nd_1__−_*_x_*Eu*_x_*NiO_2_ with *T*_c_ (e.g. peaking at ∼37.6 K). This was confirmed by higher magnitudes of *T*_c_ reaching 40.1, 38.1 and 38.4 K as observed for (Nd_0.8_Pr_0.2_)_0.65_Eu_0.35_NiO_2_, (Nd_0.6_Sm_0.4_)_0.65_Eu_0.35_NiO_2_ and (Nd_0.4_Pr_0.3_Sm_0.3_)_0.65_Eu_0.35_NiO_2_, respectively, as demonstrated by Figs S24 and S25. Considering that Nd^3+^ exhibits an ionic radius between Pr^3+^ and Sm^3+^ and a larger magnetic moment than both; the further elevation in *T*_c_ is unlikely to be related to *RE* size effect, but more associated with a weakened lattice magnetism via disturbing *RE-*4*f* spin ordering from *RE* mixing.

### Elevated *T*_c_ coincides with promoted magnetic fluctuation at superconducting state

Grounded in present large *RE*-compositional diversity, we summarized the *T*_c_ plotted as a function of the *c*-axis lattice constants (*c*) for the present Eu-doped IL nickelates showing robust superconductivity without reentrancy together with the reported ones [10,27] in Fig. 4a. This is further compared to the *T*_c_–*c* tendency developed from Sr-/Ca-doped IL nickelates approaching to optimum doping [30,31,38,44–48], as indicated by the dash line. The further elevation in *T*_c_ is clearly observed for the optimum Eu-doped IL nickelates transcending the Sr-/Ca-doped ones even at similar layer spacing. This unveils an extra pairing strengthening mechanism related to Eu^2+^-4*f* ^7^, as is further confirmed by performing magneto-electrical transport measurements shown in Fig. S26 to estimate the paring strength summarized in Table S2. It evokes closed associations with potential more complicated spin and orbital interactions with Eu^2+^-4*f* ^7^, which improves paring via potentially promoted magnetic fluctuation [49], struggling for primacy with the J–P effects.

**Figure 4.**
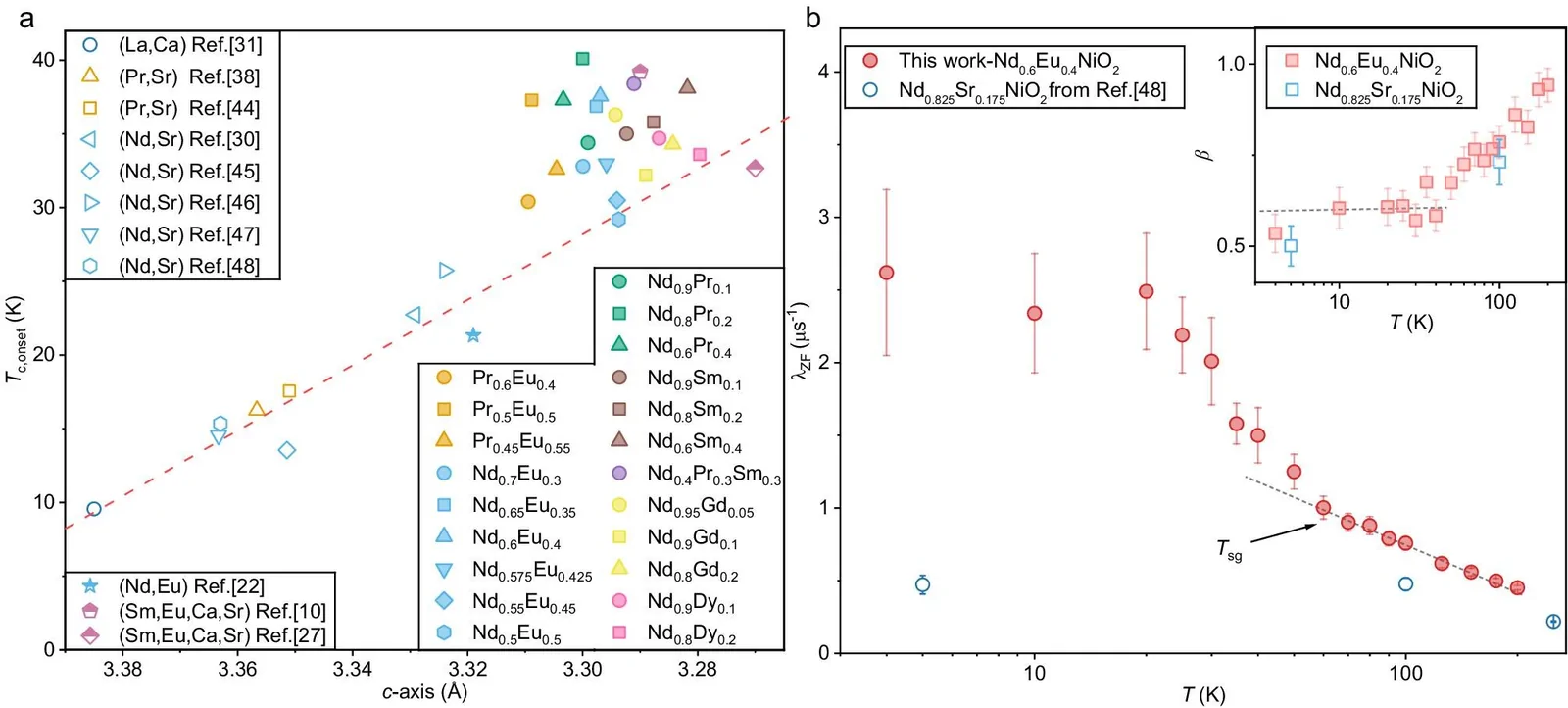
Further elevation in *T*_c_ concomitant with promoted magnetic fluctuations in Eu-doped IL nickelates. (a)The *T*_c,onset_ plotted as a function of the *c*-axis lattice constant for the present and reported [10,22,27] Eu-doped IL nickelates without reentrancy together with ones doped by Sr/Ca [30,31,38,44–48]. The dashed line is a guide to the *T*_c_–*c* trend fitted from the Sr-/Ca-doped systems. (b) The zero-field muon spin relaxation rate (*λ*_ZF_) fitted from the zero-field *μ*SR (ZF-*μ*SR) spectra plotted as a function of temperature for the optimum-doped Nd_0.6_Eu_0.4_NiO_2_ films grown on the LSAT (100) substrates, compared to the reported ones for Nd_1__−__x_Sr_x_NiO_2_ (hollow dots) [48]. Descending temperature across *T*_c_ results in abrupt elevation in *λ*_ZF_, indicating promoted spin ordering and magnetic fluctuation. The inset demonstrates the temperature dependence of the stretch exponent *β* (solid squares), showing similar temperature dependence compared with the previous report for Nd_1__−__*x*_Sr_*x*_NiO_2_ (hollow squares) [48]. The magnitude of *β* saturates to ∼0.5–0.6 below 50 K, indicating a possible spin glass-like state, as *T*_sg_ marked by the arrow.

It is also intriguing to note the abruptly promoted spin ordering below *T*_c_ even for the optimum-doped Nd_0.6_Eu_0.4_NiO_2_ with completely shielded reentrant superconductivity, as probed by LE-*μ*SR, as demonstrated in Fig. 4b. More detailed results are further shown in Figs S27 and S28. Descending temperature across 20–40 K (consistent to superconducting transition) results in an abrupt increase in the relaxation rate (λ_ZF_), accompanied by reductions in the stretch parameter *β* down to a plateau magnitude of ∼0.5–0.6 similar to spin glass [48]. The magnitude of λ_ZF_ (e.g. 2.5 μs^−1^) in the superconducting state is much larger than that previously observed for the Sr-doped IL nickelates (e.g. 0.22–0.48 μs^−1^) [48]. This illuminates a potential path to further explore associations between the promoted spin ordering in superconducting state and the significant elevation in *T*_c_ observed for Eu-doped IL nickelates (e.g. ∼37.6 K) compared to the Sr-doped ones (e.g. ∼10 K [9]).

## CONCLUSION

In conclusion, grounded in the high-*p*_O2_ chemical avenue to effectively grow (*RE*_1__−_*_y_RE'_y_*)_1__−_*_x_*Eu*_x_*NiO_2_, we elucidate the enrichment in their superconducting phase diagram by field-reentrant superconducting states emerging at both quantum boundaries, competing for primacy. The quantum criticality between reentrant and high-*T*_c_ superconductivity not only hinges on Eu composition, but also displays associations with the *RE* (*RE′*) magnetism, which modulate the exchange field together with Eu^2+^. Furthermore, combining magneto-transport and LE-*μ*SR measurements unveils the concomitant occurrence of strengthened Cooper pairing and promoted magnetic fluctuation via optimum Eu^2+^-4*f* ^7^ interactions. This counts for their further elevated *T*_c_ beyond the Sr-/Ca-doped systems even at similar IL spacing, giving rise to higher *T*_c_ up to ∼40 K and more robust *J*_c_ reaching 266 kA/cm^2^ at 2 K. These discoveries highlight a new freedom from the overlooked *RE*-4*f* orbital and spin interplays that further complicate the superconducting phase diagram of IL nickelates in terms of quantum phase criticality modulation and pairing strengthening, beyond analogous cuprates with similar *d*-orbital frameworks. Furthermore, the non-vacuum growth of IL nickelate with high effectiveness holds practical implications, particularly in potential to leverage coated conductor technologies similar to cuprates.

## METHODS

### Sample preparation

Thin films of (Nd_1__−_*_y_RE'_y_*)_1__−_*_x_*Eu*_x_*NiO_2_ (*RE'* = Pr, Sm, Gd and Dy) were grown via a high-oxygen-pressure-assisted chemical route, followed by a soft topotactic reduction. Metal nitrate precursors, such as Nd(NO_3_)_3_ and Eu(NO_3_)_3_, as well as the Ni(CH_3_COO)_2_ (≥99.9%, Aladdin) were dissolved in EGME according to the target stoichiometry. For partial *RE* substitutions, Pr(NO_3_)_3_, Sm(NO_3_)_3_, Gd(NO_3_)_3_ and Dy(NO_3_)_3_ were co-dissolved correspondingly. The precursor solution was spin-coated onto single-crystal NdGaO_3_(110) and LSAT (100) substrates and dried in air. As-deposited gel films were crystallized into perovskite-type nickelate precursors by annealing at 700–900°C under oxygen pressures of 1–20 MPa in a custom-built high-*p*_O2_ tubular reactor. After cooling to room temperature, the perovskite films were sealed in a vacuum quartz tube together with CaH_2_ powder and annealed at 260–320°C for 0.5–4 h for topotactic reduction into the IL phase. This process simultaneously reduced Eu^3+^ to Eu^2+^, serving as the effective hole dopant. The resulting IL films had a typical thickness of ∼20 nm.

### X-ray diffraction

Crystal structures of the perovskite precursors and reduced IL nickelate films were characterized using XRD (Bruker D8 Discover) with Cu *K*_α1_ radiation (λ = 1.5406 Å). The *θ*–2*θ* scans were collected to identify phase purity, and reciprocal space mapping of the (103) reflection was employed to verify the epitaxial quality and in-plane lattice coherence.

### Scanning transmission electron microscopy

The cross-sectional morphologies and interfacial structures of the samples were characterized by scanning transmission electron microscopy (STEM) using an FEI Titan Themis Z microscope operated at 300 kV, equipped with a double aberration corrector and an HAADF detector. The convergence semi-angle for imaging is 25 mrad, and the collection semi-angle is 50–200 mrad for the HAADF imaging. The cross-sectional transmission electron microscopy (TEM) lamellae were thinned to electron beam transparency at 30 kV by using Carl Zeiss Crossbeam 550 L focused ion beam-scanning electron microscopy (FIB-SEM) system, followed by the removal of the surface amorphous layer at 2 kV. The obtained HAADF-STEM images revealed atomically sharp interfaces between the Nd_1__−_*_x_*Eu*_x_*NiO_2_ films and NdGaO_3_ substrates and confirmed the topotactic structural transformation from the perovskite precursor to the IL phase after reduction. The electron energy-loss spectroscopy (EELS) and energy-dispersive X-ray spectroscopy (EDS) were performed to obtain chemical and electronic-structure information. The EELS spectrum imaging was acquired with the post-column imaging filter fitted with a K3 direct electron detector. The STEM beam current was set to ∼80 pA for spectrum imaging, the pixel dwell time was 1 ms and the recorded pixel size was 0.5 Å. The EELS energy dispersion was of 0.35 eV per channel. The EDS elemental maps were collected with an acquisition current of ∼100 pA. All EELS and EDS datasets were energy- and spatially calibrated and processed using standard routines; elemental maps shown in the manuscript were optimized in Velox software (Thermo Fisher Scientific) with a light Gaussian blur applied for presentation.

### X-ray photoelectron spectroscopy and XAS measurements

X-ray photoelectron spectroscopy (Thermo Scientific ESCALAB 250Xi) was used to determine the chemical states of Eu, Nd and Ni ions, with Al *K*_α_ (1486.6 eV) radiation. The binding energy was calibrated using the C 1*s* peak at 284.8 eV. The Eu^3+^:Eu^2+^ ratio was estimated by deconvolution of the Eu 3*d*_3/2_ and 3*d*_5/2_ peaks. The synchrotron-based XAS at the Ni *L*-edge was carried out at the BL08U1A beamline of the Shanghai Synchrotron Radiation Facility. Total electron yield mode was used to probe the near-surface region, confirming the reduction of Ni valence from +3 in Nd_0.65_Eu_0.35_NiO_3_ to +1 in Nd_0.65_Eu_0.35_NiO_2_.

### Electrical transport measurements

Electrical transport properties were measured using a Quantum Design Physical Property Measurement System (PPMS). Standard four-probe geometry was used with aluminum ultrasonic wire bonds; contact resistances were typically below a few hundred ohms. Temperature-dependent resistivity (*ρ*–*T*) measurements were performed from 300 to 2 K under magnetic fields up to 9 T. Angle-dependent transport was measured using a high-precision rotator (angular resolution 0.01°) to determine the anisotropy of reentrant superconductivity. The critical current density (*J*_c_) was measured using the Electrical Transport Option module of the same PPMS from current–voltage (*I*–*V*) curves in a four-probe configuration.

### High-field magneto-transport

High-magnetic-field resistance measurements were performed on the WM1 system at the Steady High Magnetic Field Facility, Chinese Academy of Sciences (Hefei). Magnetic fields up to 35 T and temperatures down to 1.6 K were achieved in a custom-built ^4^He cryostat (Physike Instruments). A Keithley 6221 current source and SR830 lock-in amplifiers were used for low-noise data acquisition.

### The *μ*SR measurements

The *μ*SR measurements were performed at the low-energy Muon Facility at the *μ*E4 beam line of the Swiss muon source at Paul Scherrer Institut (PSI) in Villigen, Switzerland. Implantation energies were selected from TRIM.SP simulations to maximize muon stopping rates in the Nd_0.6_Eu_0.4_NiO_2_ films. To exclude the possibility of stray magnetic field during the ZF-*μ*SR measurements, all the magnets were preliminarily degaussed, and an active field-compensating facility was used. For the weak-transverse-field (wTF) *μ*SR measurements, the applied magnetic field (i.e. 10 mT) was perpendicular to the muon-spin direction, and the films were cooled in an applied magnetic field down to the base temperature (≈1.6 K). Six pieces of Nd_0.6_Eu_0.4_NiO_2_/LSAT (100) films (each has a dimension of 5 × 10 mm^2^) were mounted on the non-magnetic Ag sample holder. The muon spin relaxation of Ag is <0.04 μs^−1^ at temperatures above 2 K, which is negligible compared with Nd_0.6_Eu_0.4_NiO_2_ films.

## Supplementary Material

nwag457_Supplemental_File

## Data Availability

The data that support the findings of this study are available from the corresponding author upon reasonable request.
