# Supplementary material for: A chemical avenue to manipulate field-reentrant superconducting competition in infinite-layer nickelates
Source: Natl Sci Rev. 2026 Jul 27;13(19):nwag457. doi: 10.1093/nsr/nwag457 (PMC13637578; doi:10.1093/nsr/nwag457)
Supplement: Supplementary file 1 [file nwag457_supplemental_file.docx]

**Supplementary Information for**

**A chemical avenue to manipulate field-reentrant superconducting** **competition in infinite layer nickelates**

Haowen Han^1,†^, Yusong Zhao^1,†^, Yi Bian^1,†^, Tong Ma^1^, Wenlong Yang^2^, Shaohua Yang^3^, Binghui Ge^3,^*, Hongliang Dong^4,5^, Chuanying Xi^6^, Ze Wang^6^, Nuofu Chen^7^, Tian Shang^8^, Toni Shiroka^9^, Zaher Salman^9^, Jia-Cai Nie^2,10,^*, Ho-Kwang Mao^4,5^, and Jikun Chen^1,^*

^1^Beijing Advanced Innovation Center for Materials Genome Engineering, School of Materials Science and Engineering, University of Science and Technology Beijing, Beijing 100083, China.

^2^School of Physics and Astronomy, Beijing Normal University, Beijing 100875, China.

^3^Institute of Physical Science and Information Technology, Anhui University, Anhui 230601, China.

^4^Center for High Pressure Science and Technology Advanced Research, Shanghai 201203, China.

^5^Shanghai Key Laboratory of Material Frontiers Research in Extreme Environments, Institute for Shanghai Advanced Research in Physical Sciences, Shanghai 201203, China.

^6^Anhui Key Laboratory of Low-Energy Quantum Materials and Devices, High Magnetic Field Laboratory, HFIPS, Chinese Academy of Sciences, Anhui 230031, China.

^7^School of Renewable Energy, North China Electric Power University, Beijing 102206, China.

^8^Key Laboratory of Polar Materials and Devices (MOE), School of Physics and Electronic Science, East China Normal University, Shanghai 200241, China.

^9^PSI Center for Neutron and Muon Sciences, Villigen PSI 5232, Switzerland.

^10^Key Laboratory of Multiscale Spin Physics, Ministry of Education, Beijing Normal University, Beijing 100875, China.

***Corresponding authors**. E-mails: [jikunchen@ustb.edu.cn](mailto:jikunchen@ustb.edu.cn); [jcnie@bnu.edu.cn](mailto:jcnie@bnu.edu.cn); [bhge@ahu.edu.cn](mailto:bhge@ahu.edu.cn)

^†^Equally contributed to this work.

**Fig. S1. Schematic of the high-pressure oxygen assisted chemical synthesis for infinite-layer nickelates and thermodynamic *p*_O2_-*T* diagram for *RE*NiO_3_.** **a,** In brief, the approach involves three steps: I, The chemical precursors were dissolved, according to their target stoichiometry. Also, other rare-earth elements, e.g., Pr, Sm, Gd and Dy, were also introduced to partially substitute Nd. As obtained solution was spin coated on NdGaO_3_(110) and LSAT(100) single crystal substrates and dried. II, A post annealing process was further performed under a high oxygen pressure (*p*_O2_) to crystallize the spin coated film into the 113-typed perovskite precursor film. III, Finally, as-obtained perovskite nickelate precursor film was annealed together with CaH_2_ powder to be topotactical reduced into infinite layer. **b-g**, The formation Gibbs free energy (Δ*G*) of *RE*NiO_3_ plotted as a function of oxygen pressure (*p*_O2_) and temperature (*T*) for *RE*NiO_3_: **b,** PrNiO_3_, **c,** NdNiO_3_, **d,** SmNiO_3_, **e,** EuNiO_3_, **f,** GdNiO_3_, **g,** DyNiO_3_. The black lines represent the *p*_O2_-*T* relationship when formation free energy (Δ*G*) equals to zero. Hence, the *RE*NiO_3_ is thermodynamically stable within upper-left region (Δ*G* <0) compared to the black line, and unstable in the bottom-right region (Δ*G*>0). It demonstrates that *RE*NiO_3_ within heavier rare-earth composition exhibits lower thermodynamic stability, requiring higher oxygen pressure to reduce their Δ*G* into negative magnitudes for synthesis.

**Details in determination of Δ*G*:** The magnitudes of Δ*G* were calculated by the following chemical reaction equation for the synthesis of *RE*NiO_3_:

$\frac{1}{2}RE_{2}O_{3}+NiO+\frac{1}{4}O_{2}=RENiO_{3}$. (1)

The thermodynamic data of LaNiO_3_ were measured by Cheng [1]: ${\Delta H}_{LNO,1000 K}$=-46.07 kJ mol^-1^，${\Delta S}_{LNO,1000 K}$=-2.64×10^-2^ kJ (K^-1^ mol^-1^). Assuming that Δ*H* and Δ*S* at 1000 K do not vary significantly with temperature within the target temperature range, the Δ*G* of LaNiO_3_ at an arbitrary temperature can be expressed as:

$\Delta G={\Delta H}_{LNO,1000 K}-T{\Delta S}_{LNO,1000 K}-\frac{1}{4}RTln(p_{O_{2}})$	(2)

, where ${\Delta H}_{LNO,1000 K}$ and ${\Delta S}_{LNO,1000 K}$ are the enthalpy change and entropy change for the formation of LaNiO_3_ at 1000 K, respectively. The *T* denotes the temperature, *R* is the ideal gas constant, and *p*_O2_ is the oxygen partial pressure. The thermodynamic trends are expected to vary linearly with the rare-earth ionic radius, which has been proved in *RE*FeO_3_ [2], *RE*CoO_3_ [3], and *RE*MnO_3_ [4]. The statistically weighted average of the slope of linear fits to enthalpy for varying the ionic radius of rare-earth (*r_RE_*) is defined as: $h\equiv\frac{d(\Delta H)}{dr_{RE}}$. Similarly, the statistically weighted average of the slope of linear fits to entropy for varying *r_RE_* is defined as: $s\equiv\frac{d(\Delta S)}{dr_{RE}}$. Accordingly, the Δ*G* of *RE*NiO_3_ can be written as [5]:

$\Delta G={\Delta H}_{LNO,1000 K}-T{\Delta S}_{LNO,1000 K}+\left( h-sT \right)(r_{RE}-r_{\mathrm{La}})-\frac{1}{4}RTln(p_{O_{2}})$	(3)

The *h* and *s* represent the magnitude in tendency for the linear variation of enthalpy and entropy with the rare-earth ionic radius, respectively, while the *r_RE_* is the 12-fold coordinated ionic radius of rare-earth. The magnitudes of *h* and *s* are determined by constructing and solving two equations based on the boundary conditions in experimental synthesis of *RE*NiO_3_ from the previous reports [6,7], (e.g., 1000 °C and 5 MPa for NdNiO_3_, 850 °C and 90 MPa for GdNiO_3_), as: *h*=20.198 kJ mol^-1^ Å^-1^, *s*=0.215 kJ K^-1^ mol^-1^ Å^-1^.

**Fig. S2. Estimating the thermodynamic stability of *RE*NiO_3_ at 800 °C.** **a,** The formation Gibbs free energy (Δ*G*) of *RE*NiO_3_ plotted as a function of oxygen pressure (*p*_O2_) at the presently used reaction temperature (*T*) of 800 °C. **b,** The required *p*_O2_ to synthesize *RE*NiO_3_ under equilibrium condition at 800 °C plotted as a function of rare-earth ionic radius (*r_RE_*). It can be seen that the increasing *p*_O2_ effectively reduces the magnitude of Δ*G* towards negative magnitudes, enabling the synthesis of *RE*NiO_3_. Also, higher *p*_O2_ is required to realize negative Δ*G* for the synthesis of *RE*NiO_3_ with heavier rare-earth composition. As already discussed in Fig. S11, the relationship between *p*_O2_ and *T* under equilibrium conditions follows:

$log\left( p_{O_{2}} \right)=\frac{4}{ln10\times RT}\left[ {\Delta H}_{LNO,1000 K}-T{\Delta S}_{LNO,1000 K}+\left( h-sT \right)(r_{RE}-r_{\mathrm{La}}) \right]$	(4)

, where ${\Delta H}_{LNO,1000 K}$ and ${\Delta S}_{LNO,1000 K}$ are the enthalpy change and entropy change for the formation of LaNiO_3_ at 1000 K, respectively. The *T* denotes the temperature, and *R* is the ideal gas constant. The *h* and *s* represent the linear variation trends of enthalpy and entropy with the rare-earth ionic radius, respectively, while *r_RE_* is the 12-fold coordinated ionic radius of rare-earth.

**Fig. S3. Excluding *RE*-compositional disproportionation in synthesis of Nd_1-x_Eu_x_NiO_3_.** Elevation in formation Gibbs free energy if Nd_1-x_Eu_x_NiO_3_ (*G*_1_) disproportionated into Nd_1-x±_*_Δ_*Eu_x±_*_Δ_*NiO_3_ (*G*_2_) at the synthetic condition for 113-typed perovskite nickelates, e.g, 800 °C under MPa-high oxygen pressure (*p*_O2_). The change in mixing entropy ($\Delta_{mix}S$) for the rare-earth co-occupation for Nd_1-x_Eu_x_NiO_3_ is estimated as [8]:

$\Delta_{mix}S=-R[xln\left( x \right)+\left( 1-x \right)\ln\left( 1-x \right)]$	(5)

, where *R* is the ideal gas constant. Further adding this entropy term into the formula for calculating the Gibbs free energy change (Δ*G*), the resultant variation in free energy via disproportionated rare-earth from Nd_1-x_Eu_x_NiO_3_ to Nd_1-x±_*_Δ_*Eu_x±_*_Δ_*NiO_3_ (*G*_2_-*G*_1_) is estimated as:

$G_{2}-G_{1}=\frac{1}{2}RT\left( \left( x-\Delta\right)\ln\left( x-\Delta\right)+\left( x+\Delta\right)\ln\left( x+\Delta\right)+\left( 1-x+\Delta\right)\ln\left( 1-x+\Delta\right)+\left( 1-x-\Delta\right)\ln\left( 1-x-\Delta\right)-2xln(x)-2(1-x)ln(1-x) \right)$. (6)

It can be seen that the disproportionated rare-earth always elevates the formation free energy (more pronounced for smaller x), which is thermodynamically less favored. This excludes the possibility for potential inhomogeneous rare-earth composition during the present MPa-high *p*_O2_ assisted chemical approach based on thermodynamic equilibrium condition, which is in contrast to plasma assisted vacuum depositions (e.g., pulsed laser deposition).

**Fig. S4. Comparing the abruption in metal-insulator transitions of *RE*NiO_3_ grown by various approaches.** The temperature dependence in the nominalized resistivity (e.g., *ρ*/*ρ*_300 K_-*T*) of *RE*NiO_3_ grown by the present MPa-high oxygen pressure assisted approach as thin films, compared with the reported ones for their vacuum deposited thin films [9-28] and bulk pellets [6,29-32]. **a,** NdNiO_3_ [6,9-15,29]; **b,** SmNiO_3_ [6,16-24,30,31]; **c,** EuNiO_3_ [25-28,32] and **d,** GdNiO_3_ [16,32]. It can be seen that the vacuum depositions such as pulsed laser deposition, magnetron sputtering, and molecular beam epitaxy, can effectively grow *RE*NiO_3_ containing light rare-earth composition (e.g., Nd). The vacuum deposited NdNiO_3_ from previous reports [9-15] as shown in **a** displays similarly abrupt resistive change across metal-insulator transitions compared to the presently used MPa-high *p*_O2_ assisted chemical approach. Compared to their bulk pellets counterparts [6,29], the NdNiO_3_ thin films coherently grown on LaAlO_3_ substrates are under biaxial compressive strains, hence displaying lower metal-insulator transition temperatures (*T*_MIT_). Nevertheless, a much more abrupt metal-insulator transition behavior was observed for SmNiO_3_ thin films grown via our MPa-high *p*_O2_ assisted chemical approach, compared to the vacuum deposited ones reported in previous literatures [16-24], as evidenced by more significant resistive switch across *T*_MIT_ shown in **b**. Further along the Lanthanide series, although there were limited report on vacuum deposited thin films of EuNiO_3_ [25-28] and GdNiO_3_ [16] as shown in **c** and **d**, respectively, their metal-insulator transition behaviors were not yet confirmed by robust temperature dependent resistivity measurements. In contrast, the EuNiO_3_ and GdNiO_3_ thin films grown by our MPa-high *p*_O2_ assisted chemical approach show robust metal-insulator transition behaviors as indicated by abrupt resistive change. The above comparisons demonstrate the advantage in our MPa-high *p*_O2_ assisted chemical approach in growing the heavier rare-earth containing perovskite nickelates.

**Fig. S5. Determination of the metal-insulator transition temperature in Nd_1-x_Eu_x_NiO_3_.** Temperature-dependent resistivity measured for Nd_1-x_Eu_x_NiO_3_ with various Eu substituting compositions of **a,** x=0.2; **b,** x=0.25; **c,** x=0.3; **d,** x=0.35; **e,** x=0.4; **f,** x=0.45; **g,** x=0.5; **h,** x=0.54; **i,** x=0.6. The insets of each figure demonstrate the temperature dependence of the temperature coefficient of resistivity (TCR). The *T*_MIT_ is determined by averaging the metal-insulator transition temperatures corresponding to the minimal value in TCR as measured during heating up and cooling down. It can be seen that all samples of Nd_1-x_Eu_x_NiO_3_ display abrupt metal-insulator transition behaviors, while the magnitude of *T*_MIT_ increases linearly with an enlarging Eu substitution ratio.

**Fig. S6. Structural evolution from Nd_1-x_Eu_x_NiO_3_ to Nd_1-x_Eu_x_NiO_2_ and the structure of Pr_1-x_Eu_x_NiO_2_.** The X-ray diffraction patterns of **a,** the Nd_1-x_Eu_x_NiO_3_ perovskite precursor and **b,** the Nd_1-x_Eu_x_NiO_2_ infinite layer at various Eu substituting compositions. It can be seen that the topotactic reduction process effectively transforms the perovskite structure of the precursor film into infinite layers. **c,** The Pr_1-x_Eu_x_NiO_2_ infinite layer at various Eu substituting compositions. **d,** The lattice parameter of *c* plotted as a function of x for Nd_1-x_Eu_x_NiO_2_ and Pr_1-x_Eu_x_NiO_2_.

**Fig. S7. Electronic structure evolution from Nd_0.65_Eu_0.35_NiO_3_ to Nd_0.65_Eu_0.35_NiO_2_.** The synchrotron-based X-ray absorption spectroscopy probing the Ni *L*-edge before and after the topotactic reduction for Nd_0.65_Eu_0.35_NiO_3_ to Nd_0.65_Eu_0.35_NiO_2_. It can be seen that the topotactic reduction decreases the relative intensity of rightwards shoulder peak of Ni-*L*_3_ edge and results in leftwards shift of the Ni-*L*_2_ edge, indicating the reduction in the valance state of Ni from Ni³⁺ to Ni^1+^. [13,33]

**Fig. S8. Morphologies of the Nd_0.7_Eu_0.3_NiO_2_ film grown on NdGaO_3_(110). a,** The low-magnification cross-section scanning transmission electron microscopy (STEM) image, showing the overall layer stack, including the top protection layers (e.g., Pt, C and Au), Nd_0.7_Eu_0.3_NiO_2_ film and NdGaO_3_ substrate. **b** and **c** STEM images at a higher magnification. **b,** The region marked in **a** by orange dashed box. **c,** The region marked in **a** by cyan dashed box. **d,** Further amplified cross-section STEM image. **e,** **f,** The representative cross-section morphologies across from film surface towards substrate. **g-i,** The representative STEM images of interfacial morphologies at different magnifications. **j-l,** The STEM images of Nd_0.7_Eu_0.3_NiO_2_ film show several representative areas of infinite-layer lattice without stacking faults. We also noticed that several previous literatures [34-36] reporting the formation of fluorites. The fluorite layers transformed from the rock salt layers via the topotactic reduction can suppress *a*-axis reorientation and promote apical oxygen deintercalation, thereby stabilizing the square-planar nickelate phase. Nevertheless, we did not observe pronounced formation of the fluorites in our case.

**Fig. S9. Elementary distribution of the Nd_0.7_Eu_0.3_NiO_2_ film grown on NdGaO_3_(110). a,** The high angle annular dark field (HAADF) image of the cross-section morphology. **b-e,** The energy dispersive X-ray spectroscopy (EDX) elemental mapping results. **b,** Eu. **c,** Nd, **d,** Ni. **e,** O. **f,** Quantitative EDX line profiles of Eu, Nd, and Ni intensities along the arrow in **a**. The Eu, Nd and Ni signals remain nearly constant throughout the film thickness. The intensity ratio of Eu, Nd and Ni is comparable with the nominal composition, confirming controlled cation incorporation and negligible interdiffusion into the substrate. **g,** The electron energy loss spectroscopy (EELS) acquisition region corresponding to the cross-section morphology with representative regions marked by solid line. **h-j,** EELS spectra extracted from regions I-VI marked in **g**. **h,** Eu-*M* edges. **i,** Nd-*M* edges. **j,** Ni-*L* edges. **k,** The EELS acquisition region corresponding to the cross-section morphology with representative regions marked by solid line. **l, m,** The EELS elemental mapping results. **l,** Eu. **m,** Nd. **n, o,** EELS spectra extracted from regions I-VIII marked in **k**. **n,** Eu-*M* edges. **o,** Nd-*M* edges. The similar line shapes and comparable relative intensities among the varied regions indicate that Eu, Nd and Ni maintain a spatially uniform chemical environment throughout the film. These EDX and EELS results reveal that the Nd_0.7_Eu_0.3_NiO_2_ film exhibits homogeneous rare-earth distribution.

**Fig. S10. Morphologies of the (Nd_0.9_Dy_0.1_)_0.65_Eu_0.35_NiO_2_ film grown on NdGaO_3_(110). a,** The low-magnification cross-section scanning transmission electron microscopy (STEM) image, showing the overall layer stack, including the top protection layers (e.g., Pt, C and Au), (Nd_0.9_Dy_0.1_)_0.65_Eu_0.35_NiO_2_ film and NdGaO_3_ substrate. **b, c,** STEM images at a higher magnification. **b,** The region marked in **a** by orange dashed box. **c,** The region marked in **a** by cyan dashed box. **d,** The representative cross-section morphologies across from film surface towards substrate. **e, f,** The representative STEM images of interfacial morphologies. **g-i,** The STEM images of (Nd_0.9_Dy_0.1_)_0.65_Eu_0.35_NiO_2_ film demonstrate several representative areas of infinite-layer lattice without stacking faults.

**Fig. S11. Elementary distribution of the (Nd_0.9_Dy_0.1_)_0.65_Eu_0.35_NiO_2_ film grown on NdGaO_3_(110). a,** The high angle annular dark field (HAADF) image of the cross-section morphology. **b-f,** The energy dispersive X-ray spectroscopy (EDX) elemental mapping results. **b,** Eu. **c,** Dy, **d,** Nd. **e,** Ni. **f,** O. **g,** Quantitative EDX line profiles of Eu, Dy, Nd and Ni intensities along the arrow in **a**. The Eu, Dy, Nd and Ni signals remain nearly constant throughout the film thickness. The intensity ratio of Eu, Nd, Dy, and Ni is comparable with the nominal composition, confirming controlled cation incorporation and negligible interdiffusion into the substrate. **h,** The electron energy loss spectroscopy (EELS) acquisition region corresponding to the cross-section morphology with representative regions marked by solid line. **i-l,** EELS spectra extracted from regions I-VII marked in **h**. **i,** Eu-*M* edges. **j,** Dy-*M* edges. **k,** Nd-*M* edges. **l,** Ni-*L* edges. The similar line shapes and comparable relative intensities among the varied regions indicate that Eu, Dy, Nd and Ni maintain a spatially uniform chemical environment throughout the film. **m,** The EELS acquisition region corresponding to the cross-section morphology with representative regions marked by solid line. (**N-P**) EELS spectra extracted from regions I-IX marked in **m**. **q,** Eu-*M* edges. **r,** Dy-*M* edges. **s,** Nd-*M* edges. The similar line shapes and comparable relative intensities among the varied regions indicate that Eu, Dy, Nd and Ni maintain a spatially uniform chemical environment throughout the film. Both EDX and EELS results demonstrate that despite the presence of some stacking fault defects, the (Nd_0.9_Dy_0.1_)_0.65_Eu_0.35_NiO_2_ film exhibits homogeneous rare-earth distribution.

**Fig. S12. Convincing the interfacial coherency for as-grown perovskite precursor films, Nd_1-x_Eu_x_NiO_3_, prior to topotactic reductions. a,** The scanning transmission electron microscopy (STEM) images of as-grown Nd_0.75_Eu_0.25_NiO_3_ on NdGaO_3_(110) show coherent interfaces, where a coherent interface is observed. **b,** Reciprocal space mapping (RSM) of the (103) plane for Nd_0.55_Eu_0.45_NiO_3_ grown on NdGaO_3_ (110) substrate, where the same in-plane reciprocal lattice vector is observed for the film and substrate. Summarizing the HAADF-STEM and RSM results for Nd_1-x_Eu_x_NiO_3_ with different Eu substitution composition convinces the coherent epitaxy of the perovskite precursor films prior to topotactic reductions.

**Fig. S13. Superconductivity and critical current density across Eu substitution in Nd_1-x_Eu_x_NiO_2_ thin films.** The temperature-dependent resistivity (*ρ*-*T*) curves for Nd_1-x_Eu_x_NiO_2_ films over a wide range of Eu substituting contents (x=0.1-0.6): **a,** x=0.1; **b,** x=0.2; **c,** x=0.25; **d,** x=0.35; **e,** x=0.4; **f,** x=0.425; **g,** x=0.5; **h,** x=0.54; **i,** x=0.6. The results for x=0.3, 0.45 and 0.52 are shown in Figure 2a, respectively in the main text. The determination of the magnitude of onset superconducting critical temperature (*T*_c,onset_) is more clearly demonstrated by the inset at the upper-left corner of each figure. The resistivity measured as a function of imparted magnetic field along the direction of *c*-axis (*ρ-B*) at low temperatures (e.g., 2-3 K) is demonstrated by the inset at the lower-right corner of each figure. It can be seen that Nd_1-x_Eu_x_NiO_2_ display superconductivity over a wide range of the Eu substituting composition within x=0.25-0.54, with zero resistance observed for x=0.35-0.54. From their *ρ-B* tendencies, a field reentrant superconductivity emerges for Nd_1-x_Eu_x_NiO_2_ with x=0.25, 0.3 and x=0.52, 0.54; and absent for x=0.35-0.5. Based on these results together with the Figure 2a shown in the main text, the superconducting phase diagram shown in Figure 2b was plotted. **j,** Temperature dependence of the critical current density (*J*_c_) for a representative superconducting Nd_1-x_Eu_x_NiO_2_ film (x=0.4), showing robust current-carrying capability throughout the superconducting state. **k,** Magnetic-field dependence of *J*_c_ measured at 2 K, showing that a substantial *J*_c_ is retained under applied field. The large *J*_c_ values and their robustness against temperature and magnetic field are consistent with the high quality and good superconducting connectivity of the films.

**Fig. S14. Identifying the emergence of reentrant superconductivity from magnetic-field dependent** **resistivity.** Resistivity measured as a function of imparted magnetic field along *c*-axis (*ρ*-*B*) measured at different temperatures for Nd_1-x_Eu_x_NiO_2_ with various Eu-substituting compositions: **a, b,** x=0.45 (optimum doped); **c,** x=0.3 (underdoped); and **d,** x=0.52 (overdoped). It can be seen that the reentrant superconductivity emerges in both underdoped Nd_0.7_Eu_0.3_NiO_2_ and overdoped Nd_0.48_Eu_0.52_NiO_2_ at low temperatures, but absent for optimum doped Nd_0.55_Eu_0.45_NiO_2_.

**Fig. S15. Identifying the emergence of reentrant superconductivity from temperature dependent resistivity at magnetic fields.** The resistivity measured as a function of temperature (*ρ*-*T*) under various magnitude of imparted magnetic fields along the *c*-axis for Nd_1-x_Eu_x_NiO_2_: **a,** displaying conventional superconductivity (x=0.45); and **b,** with reentrant superconductivity (x=0.3). The *ρ-T* curves of Nd_0.55_Eu_0.45_NiO_2_ shifted leftwards with an increasing magnitude of imparted magnetic field that monotonically suppressed conventional superconductivity. In contrast, an intriguing crossing of *ρ-T* curves is observed for increasing the magnitude of imparted magnetic field that results in reentrant superconductivity, in consistence with the observation from their *ρ-B* tendencies shown in Fig. S14.

**Fig. S16. Structural robustness and superconducting behaviour of Eu-doped infinite-layer nickelates on different substrates and rare-earth hosts.** **a,** The lattice parameter of *c* plotted as a function of x for Nd_1-x_Eu_x_NiO_2_ on LSAT(100). This reducing tendency is similar to that of Nd_1-x_Eu_x_NiO_2_ on NdGaO_3_(110) in this work as marked by the dashed line. The inset demonstrates the X-ray diffraction patterns of Nd_1-x_Eu_x_NiO_2_ on LSAT(100). **b-d,** Representative resistivity versus temperature for Nd_1-x_Eu_x_NiO_2_ thin films with Eu composition at **b,** the under-doped region (x=0.275), **c,** the optimum-doped region (x=0.4) and **d,** the over-doped (x=0.52) region. The insets show low-temperature resistivity versus magnetic field, with a red line marking the zero-resistance baseline. Magnetic field induced reentrant superconducting behaviors are observed in both underdoped and overdoped samples, and absent for the optimum doped sample. This is consistent with the observation for Nd_1-x_Eu_x_NiO_2_ on NdGaO_3_(110) as demonstrated in Fig. 2a. **e-r,** The temperature-dependent resistivity (*ρ*-*T*) curves for Pr_1-x_Eu_x_NiO_2_ films over a wide range of Eu substituting contents (x=0.2-0.775): **e,** x=0.2; **f,** x=0.25; **g,** x=0.3; **h,** x=0.35; **i,** x=0.375; **j,** x=0.4; **k,** x=0.45; **l,** x=0.5; **m,** x=0.55; **n,** x=0.6; **o,** x=0.65; **p,** x=0.7; **q,** x=0.75; **r,** x=0.775. These data show that superconductivity also emerges over a broad composition window in the Pr-based series, with field-reentrant superconductivity appearing at the underdoped and overdoped boundaries of the superconducting dome and absent near optimum doping. Compared with Nd_1-x_Eu_x_NiO_2_, the superconducting dome of Pr_1-x_Eu_x_NiO_2_ extends to higher Eu content while maintaining a similar maximum *T*_c_, supporting the generality of the competition between high-*T*_c_ and field-reentrant superconductivity in Eu-doped infinite-layer nickelates.

**Fig. S17. Estimated hole-doping in Nd_1-x_Eu_x_NiO_2_ and Pr_1-x_Eu_x_NiO_2_ and superconducting phase diagrams.** **a-c,** X-ray photoemission spectroscopy (XPS) spectra of Nd_1-x_Eu_x_NiO_2(3)_ films (x=0.52) and Pr_1-x_Eu_x_NiO_2_ (x=0.65) showing distinct features of Eu^2+^ and Eu^3+^ states. The grey curve represents the experimental data, while the colored curves are the fitting results. Quantitative analysis yields an approximate Eu^3+^:Eu^2+^ ratio of 1:1. This mixed valence provides direct evidence for the dual role of Eu in the infinite-layer nickelates. X-ray photoelectron spectroscopy (XPS) spectra of Nd_1-x_Eu_x_NiO_2(3)_ (x=0.52) and Pr_1-x_Eu_x_NiO_2_ (x=0.65) films, showing resolved Eu^2+^ and Eu^3+^ components. The grey curves represent the experimental data and the coloured curves the fitted components. Quantitative analysis indicates an Eu^2+^:Eu^3+^ ratio of approximately 1 for Nd_1-x_Eu_x_NiO_2(3)_ (x=0.52) and ~0.67 for Pr_1-x_Eu_x_NiO_2_ (x=0.65), providing direct evidence that Eu has a mixed valence and thus plays the dual role of rare-earth substitution and effective hole dopant in these infinite-layer nickelates. **d, e,** Superconducting phase diagrams of **d,** Nd_1-x_Eu_x_NiO_2_ and **e,** Pr_1-x_Eu_x_NiO_2_, replotted as a function of estimated effective hole doping derived from the XPS-determined Eu^2+^ fraction. The blue region denotes the superconducting regime. The dashed vertical line indicates the approximate hole-doping window of ~0.1-0.3 reported for Sr-substituted infinite-layer nickelates [37,38], showing that the effective hole-doping range generated by Eu^2+^ is comparable. The broader superconducting dome of the Pr-based series in nominal Eu content is therefore consistent with its lower Eu^2+^:Eu^3+^ ratio. **f,** Comparing the Eu composition triggering reentrant superconductivity in infinite layer nickelates. Solid red squares: reentrant (field-induced) regions in this work (appearing at under- and over-doped compositions); solid blue squares: optimal, non-reentrant region in this work. Open red symbols and red shading: reentrant regions reported in the literature [39-41]; open blue symbols and blue shading: non-reentrant regions reported in the literature [39-41]. It can be seen that the Eu composition of Nd_1-x_Eu_x_NiO_2_ within the present underdoped region displaying reentrancy is comparable to the overdoped ones from Nd-Eu system grown by vacuum depositions. At x=0.3=0.5, the superconductivity of our sample is robust that completely shields the exchange field interaction.

**Fig. S18. Determination of the metal–insulator transition temperature for the (Nd_1-y_*RE'*_y_)_0.65_Eu_0.35_NiO_3_ precursor films.** Temperature-dependent resistivity (*ρ-T*) as measured for (Nd_1-y_*RE'*_y_)_0.65_Eu_0.35_NiO_3_ films (*RE'*=Pr, Sm, Gd and Dy): **a-c,** y=0.1; **d,** y=0.05; **e,** y=0.1; **f,** y=0.2. The insets of each figure demonstrate the temperature dependence of the temperature coefficient of resistivity (TCR). The *T*_MIT_ is determined by averaging the metal-insulator transition temperatures correspond to the maxima in TCR as measured during heating up and cooling down. **g,** A summary of *T*_MIT_ plotted as a function of the ionic rare-earth radius (*r_RE_*). All samples display abrupt metal-insulator transition behaviors, with a generally elevating tendency in *T*_MIT_ with a reducing *r_RE_*. Some slight deviation for introducing heavy *RE* is resultant from the enlarged difference in their *r_RE_* compared to Nd, as was also observed previously [42].

**Fig. S19. Structural evolution of (Nd_1-y_*RE'*_y_)_0.65_Eu_0.35_NiO_2_. a,** The X-ray diffraction patterns of the (Nd_1-y_*RE'*_y_)_0.65_Eu_0.35_NiO_2_ infinite layer at various rare-earth substituting compositions. **b,** The lattice parameter of *c* plotted as a function of rare-earth ionic radius (*r_RE_*) for (Nd_1-y_*RE'*_y_)_0.65_Eu_0.35_NiO_2_, where a generally reducing tendency is observed in (Nd_1-y_*RE'*_y_)_0.65_Eu_0.35_NiO_2_ with smaller *r_RE_*.

**Fig. S20. Identifying the emergence of reentrant superconductivity for (Nd_1-y_*RE'*_y_)Eu_0.35_NiO_2_.** Resistivity measured as a function of imparted magnetic field along *c*-axis (*ρ*-*B*) measured at different temperatures for (Nd_1-y_*RE'*_y_)_0.65_Eu_0.35_NiO_2_ with *RE'*=Pr, Sm, Gd and Dy: **a,** (Nd_0.9_Pr_0.1_)_0.65_Eu_0.35_NiO_2_; **b,** (Nd_0.9_Sm_0.1_)_0.65_Eu_0.35_NiO_2_; **c,** (Nd_0.9_Dy_0.1_)_0.65_Eu_0.35_NiO_2_; **d,** (Nd_0.95_Gd_0.05_)_0.65_Eu_0.35_NiO_2_; **e,** (Nd_0.9_Gd_0.1_)_0.65_Eu_0.35_NiO_2_ and **f,** (Nd_0.8_Gd_0.2_)_0.65_Eu_0.35_NiO_2_. It can be seen that the reentrant superconductivity emerges for substituting Nd by Gd (*RE'*= Gd) at low temperatures, while introducing Pr, Sm, Gd and Dy to substitute Nd preserves the conventional superconductivity the same as Nd_0.65_Eu_0.35_NiO_2_.

**Fig. S21.** **Evolution in *T*_c_ for (Nd_1-y_*RE*′_y_)_0.65_Eu_0.35_NiO_2_ with *RE*′=Gd and Dy.** Comparing **a,** the *T*_c,onset_ and **b,** the *T*_c,50%_, as a function of *RE*′ substitution ratio y, where *RE*′=Gd and Dy. The starting points indicate *T*_c,onset_ and *T*_c,50%_ for Nd_0.65_Eu_0.35_NiO_2_, while the dash line indicates the ones for the top of the superconducting dome for Nd_1-x_Eu_x_NiO_2_ system (e.g., x=0.4). It can be seen that introducing Gd or Dy to partially substitute Nd results in descendance of *T*_c_, which is in contrast to the observation for *RE*′=Pr and Sm as shown in Fig. S25.

**Fig. S22. Angular-dependent evolution of the anisotropic reentrant superconductivity. a-f,** R**esistivity** measured as a function of imparted magnetic field along *c*-axis (*ρ*-*B*) measured at different temperatures with **varied out-of-plane angles *θ*** (between **magnetic field and the sample surface normal**) for (Nd_0.95_Gd_0.05_)_0.65_Eu_0.35_NiO_2_ film. **a,** ***θ***=0°, **b,** ***θ***=15°, **c,** ***θ***=45°, **d,** ***θ***=60°, **e,** ***θ***=75°, **f,** ***θ***=90° at various temperatures. The results collectively demonstrate that the field-reentrant superconducting state is strongly anisotropic and diminishes with increasing ***θ*. g,** Resistivity of (Nd_0.95_Gd_0.05_)_0.65_Eu_0.35_NiO_2_ measured for various angles between the external magnetic field and *c*-axis at 10 K. The bottom projection shows the colored mapping of the resistivity.

**Further discussions:** The anisotropic *H*_c2_ in (Nd_1-y_*RE'*_y_)_1-x_Eu_x_NiO_2_ is understood in the context of the diverse behaviors across the nickelate family, highlighting the complex role of the rare-earth elements. It is well known that Cooper pairs can be destroyed by the external magnetic field via orbital or paramagnetic depairing. Orbital depairing exhibits superconducting anisotropy, whereas paramagnetic depairing is largely isotropic as it suppresses superconductivity by the Zeeman energy costs. A central puzzle is why Nd-based nickelates (doped by Sr) are reported to have an isotropic *H*_c2_, whereas the other systems, especially La-based nickelates (doped by Sr), exhibit an anisotropic *H*_c2_, despite having a similar NiO_2_ plane structure [43,44]. It is supposed that the primary difference is the presence or absence of magnetic 4*f*-electron moments on the rare-earth site. Nd-nickelates exhibit a Kramers’ doublet ground state due to the 4*f* electrons of Nd^3+^ ions, leading to the presence of magnetic moments [43]. An external magnetic field can polarize these moments, creating a moderate internal exchange field that acts on the Cooper pairs in the NiO_2_ planes. This provides a powerful, additional paramagnetic pair-breaking channel that strongly suppresses superconductivity, thus keeping the isotropic *H*_c2_ below the Pauli limit [43,44]. In contrast, the La^3+^ ion is non-magnetic. Its lack of 4*f* moments means this additional suppressive pair-breaking channel is completely absent. Therefore, the properties observed in the La-system represent the intrinsic physics of the NiO_2_ planes (anisotropic). The Pr-nickelate (doped by Sr) is close to the La-system, since the 4*f* electrons of the Pr^3+^ ions form a singlet ground state, resulting in nonmagnetic behavior [43]. However, the role of rare-earth magnetism becomes dramatically different in systems containing strong magnetic moments, such as in Eu-doped nickelates [39,45]. The large magnetic moments of Eu^2+^/Eu^3+^ ions may introduce substantial magnetic interactions, resulting in a strong internal exchange field. And thus, (Nd_1-y_*RE'*_y_)_1-x_Eu_x_NiO_2_ can exhibit exceptionally high *H*_c2_ (most likely anisotropic) and robust field-induced re-entrant superconductivity, which can originate from an extrinsic protection mechanism, such as the Jaccarino-Peter effect [46]. These Eu^2+^/Eu^3+^ doping may give rise to a non-trivial magnetic ground state, which requires theoretical calculations and further experimental studies like resonant inelastic X-ray scattering.

**Fig. S23. Uniaxial anisotropy of the reentrant superconductivity observed for more samples.** Comparative angular magneto-transport of the reentrant superconducting phase in Gd-substituted ((Nd_0.95_Gd_0.05_)_0.65_Eu_0.35_NiO_2_) and two overdoped (Nd_0.48_Eu_0.52_NiO_2_ and Nd_0.46_Eu_0.54_NiO_2_) samples. For each sample, angular *ρ*-*B* plots and the corresponding resistivity mappings are presented, **a, b,** (Nd_0.95_Gd_0.05_)_0.65_Eu_0.35_NiO_2_; **c, d,** Nd_0.48_Eu_0.52_NiO_2_; **e, f,** Nd_0.46_Eu_0.54_NiO_2_. The data reveal that the strong uniaxial anisotropy of the reentrant state is a common feature, emerging both near the quantum critical boundary of the superconducting dome via overdoping and through the introduction of magnetic Gd^3+^ ions.

**Fig. S24. Tuning the competition between reentrant and high-*T*_c_ superconductivity within (Nd_1-y_*RE'*_y_)_1-x_Eu_x_NiO_2_ via *RE'* substitutions.** Temperature dependence of resistivity (*ρ-T*) measured for **a,** (Nd_0.9_Pr_0.1_)_0.65_Eu_0.35_NiO_2_, **b,** (Nd_0.9_Sm_0.1_)_0.65_Eu_0.35_NiO_2_, **c,** (Nd_0.9_Dy_0.1_)_0.65_Eu_0.35_NiO_2_, **d,** (Nd_0.95_Gd_0.05_)_0.65_Eu_0.35_NiO_2_, **e,** (Nd_0.9_Gd_0.1_)_0.65_Eu_0.35_NiO_2_, **f,** (Nd_0.8_Gd_0.2_)_0.65_Eu_0.35_NiO_2_, **g,** (Nd_0.8_Pr_0.2_)_0.65_Eu_0.35_NiO_2_, **h,** (Nd_0.8_Sm_0.2_)_0.65_Eu_0.35_NiO_2_, **i,** (Nd_0.8_Dy_0.2_)_0.65_Eu_0.35_NiO_2_, **j,** (Nd_0.8_Pr_0.2_)_0.55_Eu_0.45_NiO_2_, **k,** (Nd_0.8_Sm_0.2_)_0.55_Eu_0.45_NiO_2_, **l,** (Nd_0.8_Dy_0.2_)_0.55_Eu_0.45_NiO_2_, **m,** (Nd_0.8_Gd_0.2_)_0.55_Eu_0.45_NiO_2_, **n,** (Nd_0.9_Gd_0.1_)_0.5_Eu_0.5_NiO_2_, **o,** (Nd_0.9_Dy_0.1_)_0.5_Eu_0.5_NiO_2_. The magnitudes of *T*_c,onset_ are more clearly demonstrated by the upper-left insets demonstrate *ρ-T* at a zoom in temperature range. The bottom-right insets demonstrate the resistivity measured as a function of magnetic field imparted at cross-plane direction at low temperatures, from which the emergence of field reentrant superconducting behavior is judged. It can be seen that the reentrant superconducting behavior is always observed for introducing *RE'*=Gd within non-reentrant matrix at optimum Eu-doping range, but not for other rare-earth compositions (e.g., Pr, Sm and Dy), in consistency to the observation in Fig. 3.

**Fig. S25. Further elevation in *T*_c_ via co-occupation of *RE*′** **within (Nd_1-y_ *RE*′_y_)_0.65_Eu_0.35_NiO_2_. a-f,** Temperature-dependent resistivity (*ρ*-*T*) for (Nd_1-y_*RE*′_y_)_0.65_Eu_0.35_NiO_2_ with *RE*′=Pr, Sm and Pr/Sm mixture. **g, h,** Comparing g, the *T*_c,onset_ and h, the *T*_c,50%_, as a function of *RE*′ substitution ratio y, where *RE*′=Pr, Sm and Pr_1/2_Sm_1/2_. The starting points indicates *T*_c,onset_ and *T*_c,50%_ for Nd_0.65_Eu_0.35_NiO_2_, while the dash line indicates the ones for the top of the superconducting dome for Nd_1-x_Eu_x_NiO_2_ system (e.g., x=0.4). It is in particular intriguing to note that moderately introducing *RE*′, such as Sm, Pr or even their together (Sm_1/2_Pr_1/2_), to substitute Nd within the (Nd_1-y_*RE*′_y_)_0.65_Eu_0.35_NiO_2_ sheds a light on the capability to further elevate *T*_c_ beyond the top of superconducting dome for Nd_1-x_Eu_x_NiO_2_ system. Such a mixing effect is not simply related to the ionic radius of rare-earth, noticing that the average rare-earth radius is expected to be reduced via Sm substitution and enlarged via Pr substitution, both of which may increase *T*_c_. Also, both the *T*_c,50%_ and *T*_c,onset_ for Sm_1/2_Pr_1/2_ co-substituted (Nd_0.4_Pr_0.3_Sm_0.3_)_0.65_Eu_0.35_NiO_2_ (y=0.6) exceed the ones for Nd_0.6_Eu_0.4_NiO_2_ (top of the Nd_1-x_Eu_x_NiO_2_ superconducting dome). The aforementioned phenomenon evokes associations with the previous observation in elevated *T*_c_ for introducing Ca further into the Sm_1-x_Eu_x_NiO_2_ systems [47].

**Fig. S26. Variation in upper critical field revealing paring strength.** **a, b,** The temperature-dependent resistivity (*ρ*-*T*) measured for the underdoped Nd_0.725_Eu_0.275_NiO_2_ displaying reentrant superconductivity when imparting magnetic fields: **a,** in parallel to and **b,** perpendicular to the *c*-axis, up to 35 T. **c,** The temperature dependence of the upper critical field *μ*_0_*H*_c2_(*T*) obtained from **a** and **b**. The *μ*_0_*H*_c2_(*T*) is defined at 50% of the normal-state resistivity just above *T*_c,onset_, giving *μ*_0_*H*_c,⊥50%_ and *μ*_0_*H*_c,_*_∥_*_50%_ for applying fields perpendicular and parallel to the NiO_2_ planes, respectively.

We justify the use of the 50% normal-state resistivity (*R*_n_) criterion to define *μ*_0_*H*_c2_(T) as follows. In our samples exhibiting re-entrant behaviour, the intriguing crossing of *ρ-T* curves with increase of the imparted magnetic field appears only at resistivity levels about or below ≈1%*R*_n_ (see Fig. S26a and S26b for Nd_0.725_Eu_0.275_NiO_2_, see also Fig. S15) and the *ρ-T* curve crossing is distinct from the principal superconducting transition. Using such a low- resistivity criterion would conflate these anomalous features, which may be related to an internal exchange field *H*_ex_ arising from Eu^2+^ moments, with the intrinsic upper critical field and yield a non-monotonic *μ*_0_*H*_c2_(T) that is unsuitable for Ginzburg-Landau extrapolation. By contrast, the 50% *R*_n_ midpoint is a reproducible, literature-standard metric that tracks the bulk pairing transition, is minimally sensitive to local zero-resistance anomalies, and produces monotonic *μ*_0_*H*_c2_(T) for G-L fitting near *T*_c_. For these reasons *μ*_0_*H*_c2_(T) extrapolated from the 50% *R*_n_ criterion provides a robust and physically meaningful empirical gauge of pairing strength for the comparative analysis presented in Supplementary Table S2.

The Ginzburg-Landau (G-L) equations were used to fit the (critical) temperature dependence of *μ*_0_*H*_c,⊥50%_ and *μ*_0_*H*_c,_*_∥_*_50%_, as indicated by the solid lines, via:

$\mu_{0}H_{c2,\perp50\%}(T)=\frac{\Phi_{0}}{2\pi\text{ }\xi_{\mathrm{GL}}(0)^{2}}\left( 1 - \frac{T}{T_{c}} \right)$	(7)

$\mu_{0}H_{c2,\parallel50\%}\left( T \right)=\frac{\Phi_{0}}{2\pi\text{ }\xi_{\mathrm{GL}}\left( 0 \right)\text{ }d}\left( 1 - \frac{T}{T_{c}} \right)^{\frac{1}{2}}$	(8)

where *Φ*_0_ is the flux quantum, *ξ*_GL_(0) is the in-plane zero-temperature coherence length (denoted *ξ*_GL_(0) above), and *d* is the superconducting thickness. **d-f,** Analogous measurements and analysis compared to **a-c** for the optimum doped Nd_0.65_Eu_0.35_NiO_2_ displaying robust high-*T*_c_ superconductivity without reentrancy. **g, h,** Comparing the dependence of *μ*_0_*H*_c2_(*T*) plotted as a function of critical temperature under magnetic fields divided by that under zero magnetic field (*T*/*T*_c,50%_), for Nd_1-x_Eu_x_NiO_2_ system, for imparting magnetic fields both **g,** in parallel (cross-plane) and **h,** perpendicular (in-plane) to the *c*-axis. The larger magnitude in the slope or tangent near *T*_c,50%_ of the *μ*_0_*H*_c2_(*T*)-*T*/*T*_c,50%_ tendency as observed for the optimum doped sample compared to the underdoped and overdoped ones reflects more robust Cooper pairing. Extrapolated zero-temperature upper critical fields *μ*_0_*H*_c,⊥50%_(0) and *μ*_0_*H*_c,_*_∥_*_50%_(0) plotted against Eu content *x*. The values are obtained from G-L equations fit; the corresponding fit results are presented in **g** and **h**; both *μ*_0_*H*_c,⊥50%_(0) and *μ*_0_*H*_c,_*_∥_*_50%_(0) increase from the underdoped region to a maximum near the optimal *x* and decrease toward the overdoped side. Further details regarding the (Nd_0.9_Gd_0.1_)_0.65_Eu_0.35_NiO_2_ composition are provided in Supplementary Table S2. **i,** Schematic illustration for the competition between reentrant and high-*T*_c_ superconductivity within the Nd_1-x_Eu_x_NiO_2_ system. Introducing Eu not only promotes the hole doping of the infinite layer nickelates but also generates an exchange fields (e.g., *H*_ex_) counteracting with the imparted external magnetic fields (*H*_ext_). The superposition of *H*_ex_ and *H*_ext_ yields a real exchange field (*H*_real_) that potentially results in reentrant superconducting behavior, e.g., via the Jaccarino-Peter effect. Nevertheless, the meanwhile strengthened Cooper pairing at an optimum doped Eu-composition enlarges the upper critical field, as illustrated by the extension of the yellow square beyond the green one. If the upper critical field is beyond the *H*_real_, the reentrant superconductivity is shielded, and this explains the observation of reentrant superconductivity at the boundary of superconducting dome, e.g., at both the underdoped and overdoped regions.

**Fig. S27. Probing the magnetism of Nd_0.6_Eu_0.4_NiO_2_ film via low-energy muon-spin spectroscopy (*μ*SR). a**, Representative zero-field (ZF-) *μ*SR spectra collected at various temperatures between 4 K and 200 K for the optimum-doped Nd_1-x_Eu_x_NiO_2_ (x=0.4) films grown on the LSAT (100) substrates. The ZF-*μ*SR spectra were fitted to

$A_{\mathrm{ZF}}(t)=A_{0}\exp(-[\lambda_{\mathrm{ZF}}t]^{\beta})+A_{\mathrm{bg}}$ (9)

where *λ*_ZF_ represents the zero-field muon spin relaxation rate, *β* is the stretch exponent, *A*_0_ and *A*_bg_ are the initial asymmetries sensed by implanted muons in the film and sample holder (i.e., Ag), respectively. Here, we found *A*_bg_/(*A*_s_ + *A*_bg_) is close to 10% and is almost temperature-independent. **b**, Representative weak transverse-field (wTF-) μSR spectra collected at various temperatures between 4 K and 200 K in an applied magnetic field of 10 mT for Nd_0.6_Eu_0.4_NiO_2_ (x = 0.4) films. The wTF-*μ*SR spectra were fitted to

$A_{\mathrm{TF}}(t)=A_{0}e^{-\lambda_{\mathrm{TF}}t}\cos(\gamma_{\mu}Bt+\phi),$	(10)

Where *A*_0_ is the same as ZF-*μ*SR spectra, *B* is the local magnetic field sensed by implanted muons, *γ_μ_*/2π=135.53 MHz/T is the muon gyromagnetic ratio, $\phi$is the initial phase. With decreasing the temperature, the reduction of *A*_0_ and the increase of the muon-spin relaxation rate *λ*_TF_ indicate growth of local magnetic moments. **c**, The magnetic volume fraction (*F*_M_) as a function of temperature for Nd_0.6_Eu_0.4_NiO_2_ (solid dots), compared with the previous report for Nd_1-x_Sr_x_NiO_2_ (hollow dots)^48^. The *F*_M_ was calculated by:

$F_{M}(T)=1-\frac{A_{0}(T)}{A_{\mathrm{PM}}}$	(11)

The *A*_PM_ indicates the muon-spin asymmetry in the PM state. Similar magnitudes and temperature dependence in *F*_M_ are observed for the Nd_0.6_Eu_0.4_NiO_2_ and the Nd_1-x_Sr_x_NiO_2_^48^. For both ZF- and wTF-*μ*SR measurements, the muon energy is fixed at 4 keV, which was determined by the energy-scan measurements (see details in Fig. S28).

**Fig. S28. Characterization of implanting muon energy. a,** Schematic diagram depicting the sample structure, where the red arrow illustrates the incoming muon direction. **b,** Simulated muon implantation profiles for Nd_0.6_Eu_0.4_NiO_2_/(LaAlO_3_)_0.3_(Sr_2_TaAlO_6_)_0.7_(100) films performed by TRIM.SP. 4 keV is the optimal implantation energy for the Nd_0.6_Eu_0.4_NiO_2_. **c**, Asymmetry and muon-spin relaxation rate (*λ*_TF_) as a function of muon energy at 4 K and 200 K for Nd_0.4_Eu_0.4_NiO_2_ films, respectively. Both are extracted from the wTF-*μ*SR measurements (see details also in Fig. S27 in the main text).

**Table S1.** **Nominal 4f electron counts, free-ion effective magnetic moments (μ_eff_) [48] calculated from Hund’s rules/Landé** $\boldsymbol{g}_{\boldsymbol{J}}$**, and Shannon ionic radii (CN=8) [49] for the lanthanide ions relevant to this study.**
Notes: μ_eff_ values are free-ion estimates and therefore serve as nominal/reference values only; actual solid-state moments may differ because of crystal-field (CEF) effects, Van-Vleck contributions, mixed valence, and screening. Ionic radii are from Shannon (CN=8). It is also worth noticing that the impact from crystal field is not taken into consideration for the above estimation, hence the sequence of magnetic moment for ionic rare-earth may change. This is in particular the case between Gd and Dy, a larger magnetic impact in superconductivity was observed for introducing Gd in cuprates superconductors compared to Dy [50,51].

| Ion (reference valence) | 4*f* configuration | Unpaired electrons (2*S*) | Theoretical *μ*_eff_ (*μ*_B,_ free-ion, Hund) | Shannon ionic radius (Å, CN = 8) |
| --- | --- | --- | --- | --- |
| La^3+^ | 4*f*^0^ | 0 | 0.00 *μ*_B_ | 1.160 Å |
| Pr^3+^ | 4*f*^2^ | 2 (*S* = 1) | 3.58 *μ*_B_ | 1.126 Å |
| Nd^3+^ | 4*f*^3^ | 3 (*S* = 3/2) | 3.62 *μ*_B_ | 1.109 Å |
| Sm^3+^ | 4*f*^5^ | 5 (*S* = 5/2) | 3.58 *μ*_B_ | 1.079 Å |
| Eu^3+^ | 4*f*^6^ | 6 (*S* = 3) | ≈ 0.00 *μ*_B_ ^*^ | 1.066 Å |
| Eu^2+^ | 4*f*^7^ | 7 (*S* = 7/2) | 7.94 *μ*_B_ | 1.250 Å |
| Gd^3+^ | 4*f*^7^ | 7 (*S* = 7/2) | 7.94 *μ*_B_ | 1.053 Å |
| Dy^3+^ | 4*f*^9^ | 5 (*S* = 5/2) | ≈ 10.65 *μ*_B_ | 1.027 Å |

* Solid-state Van-Vleck/excited-state contributions often give nonzero apparent *μ*_eff_, typically reported ≈2-4 *μ*_B_

**Calculation of theoretical effective magnetic moment (free-ion estimate)**

The effective magnetic moment for a free ion in the Russell-Saunders (LS) coupling limit is calculated from the Landé $g_{J}$factor and the total angular momentum $J$. The relevant formulae are:

$$g_{J} = 1 + \frac{J*\left( J+1 \right)+S*\left( S+1 \right)-L*(L+1)}{2*J*(J+1)}$$

$$\mu_{\mathrm{eff}}=\text{ }g_{J}\text{ }\sqrt{J*\left( J+1 \right)}\text{ }\mu_{B}$$

where $L$and $S$are the total orbital and spin angular-momentum quantum numbers (obtained from Hund’s rules for the given $4f$configuration), $J$is the total angular momentum (for less-than-half-filled shells $J=\mid L-S\mid$; for more-than-half-filled shells $J=L+S$), and $\mu_{B}$is the Bohr magneton. For purely spin systems (effective $L\approx0$), the spin-only form reduces to

$$\mu_{\mathrm{eff}}=g_{S}\text{ }\sqrt{S*\left( S+1 \right)}\text{ }\mu_{B}$$

**Worked examples (step-by-step arithmetic)**

1. **Gd^3+^ (4*f*^7^, half-filled,** $L=0$**,** $S=7/2$**,** $J=7/2$**)**

- Compute $J(J+1)$and $S(S+1)$:

$$J(J+1)=15.75$$

$$S(S+1)=15.75$$

- Landé factor:

$$g_{J}=2$$

- Effective moment:

$$\mu_{\mathrm{eff}}=2\times\sqrt{15.75}\text{ }\mu_{B}$$

$$\mu_{\mathrm{eff}}\approx7.94\text{ }\mu_{B}$$

2. **Nd^3+^ (4*f*^3^,** $L=6$**,** $S=3/2$**,** $J=9/2$**)**

- Compute angular-momentum products:

$$J(J+1)=24.75$$

$$S(S+1)=3.75$$

$$L(L+1)=42$$

- Landé factor:

$$g_{J}\approx0.727$$

- Effective moment:

$$\mu_{\mathrm{eff}}=0.73\times\sqrt{24.75}\text{ }\mu_{B}$$

$$\mu_{\mathrm{eff}}\approx3.62\text{ }\mu_{B}.$$

Theoretical (free-ion) effective magnetic moments $\mu_{\mathrm{eff}}$reported in Table S1 were computed using the Landé $g_{J}$factor and the relation $\mu_{\mathrm{eff}}=g_{J}\sqrt{J*(J+1)}\text{ }\mu_{B}$, with $L$, $S$and $J$assigned according to Hund’s rules for the given $4f$configuration. Values are free-ion estimates; solid-state moments may differ because of crystal-field splitting, Van-Vleck contributions, covalency and screening. Representative worked examples for Nd^3+^ and Gd^3+^ are given in the Supplementary Methods.

**Table S2. Ginzburg-Landau-extrapolated upper critical fields and normalized *μ*_0_*H*_c2_(0)/*****T*_c,50%_ for samples.** *μ*_0_*H*_c,⊥50%_(0) and *μ*_0_*H*_c,_*_∥_*_50%_(0) are the in-plane and out-plane upper critical fields extrapolated to 0 K using Ginzburg-Landau fits. The normalized quantity *μ*_0_*H*_c2_(0)/*T*_c,50%_ is in units of T/K.

| Samples | *T*_c,50%_ (K) | *μ*_0_*H*_c,⊥50%_(0) (T) | *μ*_0_*H*_c,_*_∥_*_50%_(0) (T) | *μ*_0_*H*_c,⊥50%_(0)/ *T*_c,50%_ (T/K) | *μ*_0_*H*_c,_*_∥_*_50%_(0)/ *T*_c,50%_ (T/K) |
| --- | --- | --- | --- | --- | --- |
| Nd_0.725_Eu_0.275_NiO_2_ | 22 | 81.9 | 67.8 | 3.7 | 3.1 |
| Nd_0.7_Eu_0.3_NiO_2_ | 22.4 | 99.6 | 92.8 | 4.4 | 4.1 |
| Nd_0.65_Eu_0.35_NiO_2_ | 27 | 146.7 | 117.5 | 5.4 | 4.4 |
| Nd_0.6_Eu_0.4_NiO_2_ | 27.3 | 113.3 | 121.0 | 4.2 | 4.4 |
| Nd_0.48_Eu_0.52_NiO_2_ | 17 | 63.5 | 35.5 | 3.7 | 2.1 |
| (Nd_0.9_Gd_0.1_)_0.65_Eu_0.35_NiO_2_ | 24.2 | 68.9 | 51.3 | 2.9 | 2.1 |

We justify the use of the 50% normal-state resistivity (*R*_n_) criterion to define *μ*_0_*H*_c2_(T) as follows. In our samples exhibiting re-entrant behaviour, the intriguing crossing of *ρ-T* curves with increase of the imparted magnetic field appears only at resistivity levels about or below ≈1%*R*_n_ (see Fig. S26a and S26b for Nd_0.725_Eu_0.275_NiO_2_, see also Fig. S15) and the *ρ-T* curve crossing is distinct from the principal superconducting transition. Using such a low-resistivity criterion would conflate these anomalous features, which may be related to an internal exchange field *H*_ex_ arising from Eu^2+^ moments, with the intrinsic upper critical field and yield a non-monotonic *μ*_0_*H*_c2_(T) that is unsuitable for Ginzburg-Landau extrapolation. By contrast, the 50% *R*_n_ midpoint is a reproducible, literature-standard metric that tracks the bulk pairing transition, is minimally sensitive to local zero-resistance anomalies, and produces monotonic *μ*_0_*H*_c2_(T) for G-L fitting near *T*_c_. For these reasons *μ*_0_*H*_c2_(T) extrapolated from the 50% *R*_n_ criterion provides a robust and physically meaningful empirical gauge of pairing strength for the comparative analysis presented in Table S2.

The values of *μ*_0_*H*_c2_(0) reported in Table S2 are Ginzburg-Landau extrapolations. To assess the sensitivity of the superconducting state to paramagnetic pair breaking we use the normalized metric *μ*_0_*H*_c2_(0)/*T*_c,50%_ as an empirical measure of the pairing energy scale (cf. the Pauli-limit 1.86 T/K). It should be noted that all *μ*_0_*H*_c2_(0)/*T*_c,50%_ ratios exceed this weak-coupling Pauli reference, indicating strongly correlated and unconventional superconductivity in the (Nd_1-y_*RE'*_y_)_1-x_Eu_x_NiO_2_. The optimally doped compositions, Nd_0.65_Eu_0.35_NiO_2_ and Nd_0.6_Eu_0.4_NiO_2_, exhibit the largest *μ*_0_*H*_c,⊥50%_(0)/*T*_c,50%_(≈5.4 and 4.2 T/K, respectively), indicating that the energy scale of pairing substantially exceeds the Pauli-limit and thus endowing the condensate robust against magnetic pair-breaking [52]. By contrast, the under- and over-doped compositions (Nd_0.725_Eu_0.275_NiO_2_ and Nd_0.48_Eu_0.52_NiO_2_) show markedly reduced *μ*_0_*H*_c2_(0) (*e.g.*, 67.8 T and 35.5 T for *μ*_0_*H*_c,_*_∥_*_50%_(0), respectively, which could be smaller than the internal exchange field-*H*_ex_), consistent with weaker pairing and hence greater vulnerability to paramagnetic perturbations [52]. This provides a natural explanation for the re-entrant behaviour observed in these compositions. The (Nd_0.9_Gd_0.1_)_0.65_Eu_0.35_NiO_2_ sample exhibits distinct yet relatively small in-plane and out-of-plane values (51.3 T and 68.9 T for *μ*_0_*H*_c,_*_∥_*_50%_(0) and *μ*_0_*H*_c,⊥50%_(0), respectively). At low temperatures, the reentrant superconducting phenomena could be observed in both directions, demonstrating that pair breaking is not only temperature-dependent but also direction-dependent and explaining the axis-selective manifestation of re-entrance. In short, when *μ*_0_*H*_c2_(0) is high the superconducting condensate is intrinsically robust and re-entrance is suppressed; when *μ*_0_*H*_c2_(0) is reduced (either by off-optimal doping or by compositional substitution that weakens pairing in a given crystallographic direction), paramagnetic pair breaking can dominate and re-entrant superconductivity emerges. (See Fig. S26 for the full numerical values and G-L-fit details.)

**References**

1.	Cheng J, Navrotsky A, Zhou X-D *et al.* Enthalpies of formation of LaMO_3_ perovskites (M = Cr, Fe, Co, and Ni). *Journal of Materials Research* 2005; **20**: 191-200.

2.	Y.Tretyakov, Kaul A, Portnoy V. Formation of rare earth and yttrium orthoferrites: A thermodynamic study. *High Temperature Science* 1977; **9**: 61-70.

3.	Petrov AN, Cherepanov VA, Zuyev AY *et al.* Thermodynamic stability of ternary oxides in LnMO_3_ (Ln = La, Pr, Nd; M = Co, Ni, Cu) systems. *Journal of Solid State Chemistry* 1988; **77**: 1-14.

4.	Atsumi T, Ohgushi T, Kamegashira N. Studies on oxygen dissociation pressure of LnMnO_3_ (Ln = rare earth) with the e.m.f. technique. *Journal of Alloys and Compounds* 1996; **238**: 35-40.

5.	Jaramillo R, Schoofs F, Ha SD *et al.* High pressure synthesis of SmNiO_3_ thin films and implications for thermodynamics of the nickelates. *Journal of Materials Chemistry C* 2013; **1**: 2455-2462.

6.	Escote MT, da Silva AML, Matos JR *et al.* General Properties of Polycrystalline LnNiO3 (Ln=Pr, Nd, Sm) Compounds Prepared through Different Precursors. *Journal of Solid State Chemistry* 2000; **151**: 298-307.

7.	Alonso JA, Martínez-Lope MJ, Casais MT *et al.* High-Pressure Preparation, Crystal Structure, Magnetic Properties, and Phase Transitions in GdNiO_3_ and DyNiO_3_ Perovskites. *Chemistry of Materials* 1999; **11**: 2463-2469.

8.	Kumar S, Jagielski J, Tian T *et al.* Mixing Entropy-Induced Layering Polydispersity Enabling Efficient and Stable Perovskite Nanocrystal Light-Emitting Diodes. *ACS Energy Letters* 2019; **4**: 118-125.

9.	Hauser AJ, Mikheev E, Moreno NE *et al.* Correlation between stoichiometry, strain, and metal-insulator transitions of NdNiO_3_ films. *Applied Physics Letters* 2015; **106**.

10.	Shao T, Qi Z, Wang Y *et al.* Metal-insulator transition in epitaxial NdNiO_3_ thin film: A structural, electrical and optical study. *Applied Surface Science* 2017; **399**: 346-350.

11.	Guo Q, Farokhipoor S, Magén C *et al.* Tunable resistivity exponents in the metallic phase of epitaxial nickelates. *Nature Communications* 2020; **11**: 2949.

12.	Liu J, Kargarian M, Kareev M *et al.* Heterointerface engineered electronic and magnetic phases of NdNiO_3_ thin films. *Nature Communications* 2013; **4**: 2714.

13.	Parzyck CT, Gupta NK, Wu Y *et al.* Absence of 3*a*_0_ charge density wave order in the infinite-layer nickelate NdNiO_2_. *Nature Materials* 2024; **23**: 486-491.

14.	Catalan G, Bowman RM, Gregg JM. Metal-insulator transitions in NdNiO_3_ thin films. *Physical Review B* 2000; **62**: 7892-7900.

15.	Laffez P, Zaghrioui M, Monot I *et al.* Microstructure and metal–insulator transition of NdNiO_3_ thin films on various substrates. *Thin Solid Films* 1999; **354**: 50-54.

16.	Wang L, Stoerzinger KA, Chang L *et al.* Tuning Bifunctional Oxygen Electrocatalysts by Changing the A-Site Rare-Earth Element in Perovskite Nickelates. *Advanced Functional Materials* 2018; **28**: 1803712.

17.	Ambrosini A, Hamet J-F. Sm_x_Nd_1−x_NiO_3_ thin-film solid solutions with tunable metal–insulator transition synthesized by alternate-target pulsed-laser deposition. *Applied Physics Letters* 2003; **82**: 727-729.

18.	Conchon F, Boulle A, Guinebretière R *et al.* Effect of tensile and compressive strains on the transport properties of SmNiO_3_ layers epitaxially grown on (001) SrTiO_3_ and LaAlO_3_ substrates. *Applied Physics Letters* 2007; **91**.

19.	Girardot C, Pignard S, Weiss F *et al.* SmNiO_3_/NdNiO_3_ thin film multilayers. *Applied Physics Letters* 2011; **98**.

20.	Ha SD, Vetter U, Shi J *et al.* Electrostatic gating of metallic and insulating phases in SmNiO_3_ ultrathin films. *Applied Physics Letters* 2013; **102**.

21.	Shukla N, Joshi T, Dasgupta S *et al.* Electrically induced insulator to metal transition in epitaxial SmNiO_3_ thin films. *Applied Physics Letters* 2014; **105**.

22.	Xiang P-H, Asanuma S, Yamada H *et al.* Room temperature Mott metal-insulator transition and its systematic control in Sm_1−x_Ca_x_NiO_3_ thin films. *Applied Physics Letters* 2010; **97**.

23.	Shi J, Ha SD, Zhou Y *et al.* A correlated nickelate synaptic transistor. *Nature Communications* 2013; **4**: 2676.

24.	Shi J, Zhou Y, Ramanathan S. Colossal resistance switching and band gap modulation in a perovskite nickelate by electron doping. *Nature Communications* 2014; **5**: 4860.

25.	Taniguchi Y, Li H-B, Shimoyama K *et al.* Epitaxial growth of EuNiO_3_ on SrTiO_3_ and its application to stacked protonation resistance switching devices. *Applied Physics Letters* 2023; **122**.

26.	Muzzillo CP, Sahebkar K, Smeaton MA *et al.* Epitaxial stabilization and oxygen vacancy control of EuNiO_3_ thin films. *Journal of Applied Physics* 2025; **138**.

27.	Bilewska K, Wolna E, Edely M *et al.* Evidence of charge disproportionation on the nickel sublattice in EuNiO_3_ thin films: X-ray photoemission studies. *Physical Review B* 2010; **82**: 165105.

28.	Meyers D, Middey S, Kareev M *et al.* Strain-modulated Mott transition in EuNiO_3_ ultrathin films. *Physical Review B* 2013; **88**: 075116.

29.	García-Muñoz JL, Aranda MAG, Alonso JA *et al.* Structure and charge order in the antiferromagnetic band-insulating phase of NdNiO_3_. *Physical Review B* 2009; **79**: 134432.

30.	Nikulin IV, Novojilov MA, Kaul AR *et al.* Synthesis and transport properties study of Nd_1−x_Sm_x_NiO_3−_*_δ_* solid solutions. *Materials Research Bulletin* 2004; **39**: 803-810.

31.	Torrance JB, Lacorre P, Nazzal AI *et al.* Systematic study of insulator-metal transitions in perovskites RNiO_3_ (R=Pr,Nd,Sm,Eu) due to closing of charge-transfer gap. *Physical Review B* 1992; **45**: 8209-8212.

32.	Li Z, Yan F, Li X *et al.* Molten-salt synthesis of rare-earth nickelate electronic transition semiconductors at medium high metastability. *Scripta Materialia* 2022; **207**: 114271.

33.	Nie J, Chen X, Bian Y *et al.* Irrelevance of ^1^H composition to superconductivity in infinite-layer nickelates from measurements of nuclear interactions. *Newton* 2025; **1**.

34.	Onozuka T, Chikamatsu A, Katayama T *et al.* Formation of defect-fluorite structured NdNiO_x_H_y_ epitaxial thin films via a soft chemical route from NdNiO_3_ precursors. *Dalton Transactions* 2016; **45**: 12114-12118.

35.	Ferenc Segedin D, Goodge BH, Pan GA *et al.* Limits to the strain engineering of layered square-planar nickelate thin films. *Nature Communications* 2023; **14**: 1468.

36.	Yan X, Zheng H, Li Y *et al.* Superconductivity in an ultrathin multilayer nickelate. *Science Advances* 2025; **11**: eado4572.

37.	Lee K, Wang BY, Osada M *et al.* Linear-in-temperature resistivity for optimally superconducting (Nd,Sr)NiO_2_. *Nature* 2023; **619**: 288-292.

38.	Osada M, Wang BY, Lee K *et al.* Phase diagram of infinite layer praseodymium nickelate Pr_1−x_Sr_x_NiO_2_ thin films. *Physical Review Materials* 2020; **4**.

39.	Wei W, Vu D, Zhang Z *et al.* Superconducting Nd_1-x_Eu_x_NiO_2_ thin films using in situ synthesis. *Science Advances* 2023; **9**: eadh3327.

40.	Vu D, Lee H, Nicoletti D *et al.* Re-entrant unconventional superconductivity induced by rare-earth substitution in Nd_1-_*_x_*Eu*_x_*NiO_2_ thin films. *Nature Communications* 2026.

41.	Yang M, Tang J, Wu X *et al.* Robust field re-entrant superconductivity in ferromagnetic infinite-layer rare-earth nickelates. *arXiv 250814666* 2025.

42.	Li Z, Li X, Zhang F *et al.* Metal-to-insulator transition and stability for metastable correlated perovskite nickelates with complex *RE*-composition. *Journal of the American Ceramic Society* 2025; **108**: e70064.

43.	Wang BY, Wang TC, Hsu Y-T *et al.* Effects of rare-earth magnetism on the superconducting upper critical field in infinite-layer nickelates. *Science Advances* 2023; **9**: eadf6655.

44.	Wang BY, Li D, Goodge BH *et al.* Isotropic Pauli-limited superconductivity in the infinite-layer nickelate Nd_0.775_Sr_0.225_NiO_2_. *Nature Physics* 2021; **17**: 473-477.

45.	Rubi K, Yip KY, Krenkel E *et al.* Extreme magnetic field-boosted superconductivity in a high-temperature superconductor. *arXiv preprint arXiv:250816290* 2025.

46.	Meul HW, Rossel C, Decroux M *et al.* Observation of Magnetic-Field-Induced Superconductivity. *Physical Review Letters* 1984; **53**: 497-500.

47.	Yang M, Wang H, Tang J *et al.* Enhanced superconductivity and mixed-dimensional behaviour in infinite-layer samarium nickelate thin films. *Nature Communications* 2026; **17**: 2761.

48.	Abragam A, Bleaney B. Electron paramagnetic resonance of transition ions: OUP oxford, 2012.

49.	Shannon R. Revised effective ionic radii and systematic studies of interatomic distances in halides and chalcogenides. *Acta Crystallographica Section A* 1976; **32**: 751-767.

50.	Xu Y, Guan W. Ion-size effect on *T*_c_ in (*R*_1-_*_x_*Pr*_x_*)Ba_2_Cu_3_O_7-_*_y_* systems (*R*=Nd, Eu, Gd, Dy, Y, Er, and Yb). *Physical Review B* 1992; **45**: 3176.

51.	Charoenthai N, Suryanarayanan R, Varamit S *et al.* Structural Changes and Suppression of Superconductivity in Two Re.

52.	Osada M, Imajo S, Seki Y *et al.* Systematic evolution of superconducting pairing strength and Seebeck coefficients in correlated infinite-layer La_1-x_Sr_x_NiO_2_. *Science Advances* 2025; **11**: eadv6488.
